# Interleukin-7 receptor α mutational activation can initiate precursor B-cell acute lymphoblastic leukemia

**DOI:** 10.1038/s41467-021-27197-5

**Published:** 2021-12-14

**Authors:** Afonso R. M. Almeida, João L. Neto, Ana Cachucho, Mayara Euzébio, Xiangyu Meng, Rathana Kim, Marta B. Fernandes, Beatriz Raposo, Mariana L. Oliveira, Daniel Ribeiro, Rita Fragoso, Priscila P. Zenatti, Tiago Soares, Mafalda R. de Matos, Juliana Ronchi Corrêa, Mafalda Duque, Kathryn G. Roberts, Zhaohui Gu, Chunxu Qu, Clara Pereira, Susan Pyne, Nigel J. Pyne, Vasco M. Barreto, Isabelle Bernard-Pierrot, Emannuelle Clappier, Charles G. Mullighan, Ana R. Grosso, J. Andrés Yunes, João T. Barata

**Affiliations:** 1 Instituto de Medicina Molecular João Lobo Antunes, Faculdade de Medicina, Universidade de Lisboa, Lisbon, Portugal; 2 Centro Infantil Boldrini, Campinas, SP Brazil; 3 Institut Curie, PSL Research University, CNRS, UMR144, Equipe Labellisée Ligue contre le Cancer, Paris, France; 4 Hematology Laboratory, Saint-Louis Hospital, AP-HP, Paris, France, and Saint-Louis Research Institute, Université de Paris, INSERM U944/Centre National de la Recherche Scientifique (CNRS) Unité Mixte de Recherche (UMR) 7212, Paris, France; 5 Department of Pathology and Hematological Malignancies Program, St. Jude Children’s Research Hospital, Memphis, TN US; 6 Smurfit Institute of Genetics, Trinity College Dublin, University of Dublin, Dublin 2, Ireland; 7 Strathclyde Institute of Pharmacy and Biomedical Sciences (SIPBS), University of Strathclyde, Glasgow, Scotland UK; 8 DNA Breaks Laboratory, CEDOC - Chronic Diseases Research Center, NOVA Medical School - Faculdade de Ciências Médicas, Universidade NOVA de Lisboa, Lisbon, Portugal; 9 UCIBIO, Departamento de Ciências da Vida, Faculdade de Ciências e Tecnologia, Universidade NOVA de Lisboa, Caparica, Portugal

**Keywords:** Cancer models, Acute lymphocytic leukaemia, Oncogenes, Molecular medicine, Acute lymphocytic leukaemia

## Abstract

Interleukin-7 receptor α (encoded by *IL7R*) is essential for lymphoid development. Whether acute lymphoblastic leukemia (ALL)-related *IL7R* gain-of-function mutations can trigger leukemogenesis remains unclear. Here, we demonstrate that lymphoid-restricted mutant *IL7R*, expressed at physiological levels in conditional knock-in mice, establishes a pre-leukemic stage in which B-cell precursors display self-renewal ability, initiating leukemia resembling PAX5 P80R or Ph-like human B-ALL. Full transformation associates with transcriptional upregulation of oncogenes such as *Myc* or *Bcl2*, downregulation of tumor suppressors such as *Ikzf1* or *Arid2*, and major IL-7R signaling upregulation (involving JAK/STAT5 and PI3K/mTOR), required for leukemia cell viability. Accordingly, maximal signaling drives full penetrance and early leukemia onset in homozygous *IL7R* mutant animals. Notably, we identify 2 transcriptional subgroups in mouse and human Ph-like ALL, and show that dactolisib and sphingosine-kinase inhibitors are potential treatment avenues for IL-7R-related cases. Our model, a resource to explore the pathophysiology and therapeutic vulnerabilities of B-ALL, demonstrates that *IL7R* can initiate this malignancy.

## Abstract (web summary)

Interleukin-7 receptor alpha (IL7Ra) is important for lymphoid cell development but its role in leukaemogenesis is not clear. Here, the authors generate a knock-in murine model to show that activating mutations in IL7Ra can initiate precursor B-cell acute lymphoblastic leukaemia.

## Introduction

Acute lymphoblastic leukemia (ALL), the most common childhood malignancy, is an aggressive cancer arising from lymphoid progenitors, especially of the B lineage^1^. Although current therapies are highly effective, with 5-year survival rates reaching 80–90%, a significant number of ALL cases still relapse and the use of intensive chemotherapy has substantial short- and long-term side effects, including decreased life expectancy^1,2^. Importantly, therapeutic success in adults lags significantly behind, with only 30–40% of the cases surviving long term^3^. As such, improving the understanding of ALL molecular causes and underlying biology is critical to better classify patients and identify the best targeted treatment options that can improve efficacy and minimize toxicities for each particular patient subset.

The axis constituted by interleukin-7 (IL-7) and its receptor (IL-7R), composed of IL-7Rα (encoded by *IL7R*) and γc (encoded by *IL2RG*), is essential for normal lymphoid development—its inactivation resulting in severe combined immunodeficiency^4–7^. During B cell development, IL-7Rα signaling is tightly regulated and, together with the pre-BCR, sequentially coordinates proliferation and *Ig* gene rearrangements, in cross-talk with critical transcription factors such as PAX5, EBF1 or IKAROS^8,9^. The importance of keeping IL-7R-mediated signaling under control is illustrated by studies showing that *Il7* transgenic mice develop B cell lymphomas, and that IL-7 induces proliferation of human B-ALL cells^7^. Moreover, B-ALL arising in *Pax5*-deficient mice exposed to infection^10^ or in mice with combined loss of *Sh2b3* and *Trp53* (ref. ^11^) involves an IL-7 hypersensitive pre-leukemic stage, and IL-7Rα is required for B-ALL developing in mice from the combination of *Stat5* activation and *Pax5* haploinsufficiency^12^.

Somatic *IL7R* gain-of-function mutations were identified in around 10% of T-ALL cases and in B-ALL^13,14^, where they are enriched in particular subgroups, including Ph-like and PAX5 P80R B-ALL^15–19^. Studies relying on retroviral transduction of mouse hematopoietic progenitors or thymocytes and subsequent transplantation into recipient mice showed that overexpression of mutant IL-7Rα can collaborate with other oncogenic insults (*Cdkn2a* deletion, *NRAS* G13D mutation, and intracellular NOTCH1 overexpression) to promote T-ALL^20–22^. These studies, however, did not separate the effect of *IL7R* mutation from *IL7R* overexpression, and were conducted in the context of already compromised hematopoiesis (in immunodeficient or irradiated recipient mice). Moreover, ectopic *IL7R* expression can considerably bias the results, as illustrated by mutant IL-7Rα resulting in myeloid neoplasia or IgM-positive mature B cell leukemia/lymphoma^22^, for which no *IL7R* mutations have been reported in humans. Thus, it remains unclear whether mutational activation of IL-7Rα without concomitant overexpression, can effectively trigger ALL, i.e., whether *IL7R* mutations per se can be the initiating event. This is particularly relevant because it is commonly accepted that ALL is triggered by fusions (such as BCR-ABL1, ETV6-RUNX1 or those involving KMT2A) resulting from chromosomal rearrangements^23^, or by lesions affecting transcription factors (such as PAX5 P80R)^16^, but there is no clear evidence that gain-of-function mutations in signaling-related genes can initiate ALL.

Here, we generated and characterized a conditional knock-in model in which mutant IL-7Rα is expressed at physiological levels from the common lymphoid progenitor (CLP) stage. We demonstrate that *IL7R* gain-of-function is sufficient to trigger leukemogenesis leading to the development of PAX P80R or Ph-like precursor B-ALL with high penetrance, reflecting the enrichment in these two subtypes within *IL7R* mutant human cases. We find that IL7R-dependent Ph-like ALL in the mouse subdivides into two subgroups with distinct transcriptional and mutational signatures that also exist in human cases. Subsequent hits potentially contributing to leukemia development affect genes known to be involved in human B-ALL (e.g *Pax5*, *Trp53*, *Kras*) as well as new candidates (e.g *Limk1*, *Cdc42bpb*). We further show that *Kras* mutation cooperates with *IL7R* mutation in upregulating IL7R-mediated signaling in pre-leukemic cells. In addition, leukemias display transcriptional upregulation of oncogenes such as *Myc* or *Bcl2*, and downregulation of tumor suppressors such as *Ikzf1*, *Ikzf2*, *Arid1b*, or *Arid2*. Irrespective of the collaborating hits, the transformation process clearly associates with a striking increase in IL-7R-mediated signaling (evidenced e.g. by mTOR and STAT5 signaling upregulation, as well as MYC activation) from the pre-leukemic to the leukemic stage, and IL-7R-mediated signaling is required for leukemia cell maintenance. Homozygous *IL7R* mutant mice, which show maximal signaling, develop very rapid, fatal leukemia with a trend for lower mutational burden, thereby illustrating the importance of IL-7R signaling upregulation in driving leukemogenesis. Finally, we use our model and human patient samples to demonstrate that PI3K/mTOR and sphingosine kinase inhibitors may constitute valid therapeutic approaches to treat *IL7R* mutant ALL cases.

## Results

### Physiological levels of heterozygous mutant IL-7Rα expression consistently originate a B cell precursor pre-leukemic stage

**Fig. 1:**
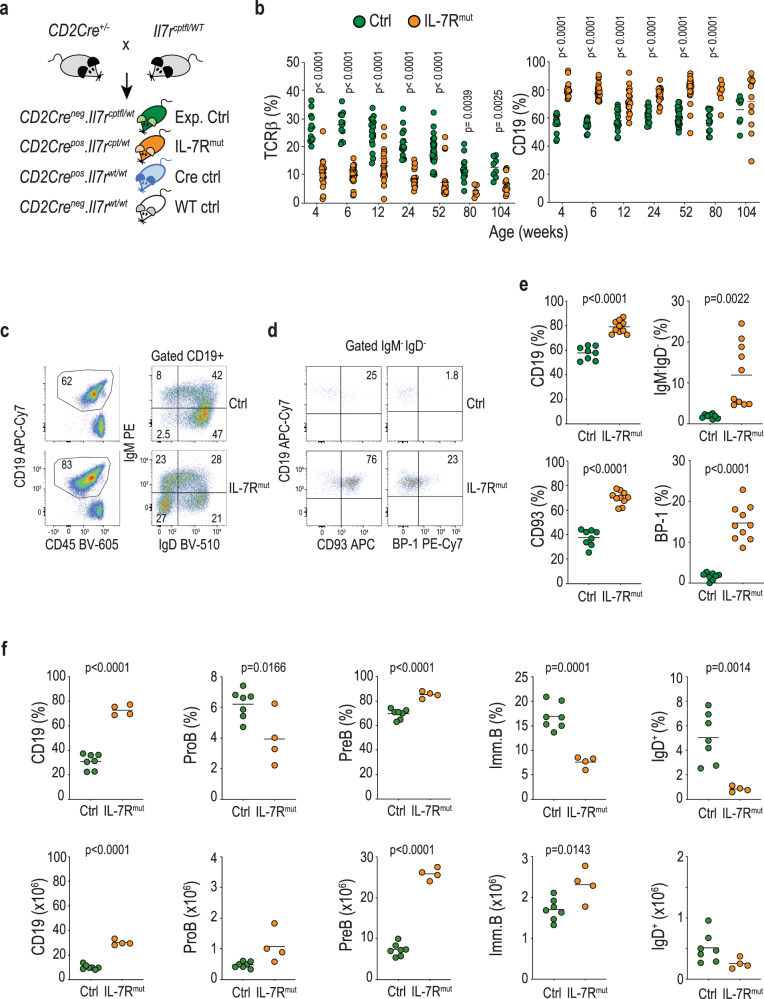
Physiological levels of heterozygous mutant IL-7Rα expression consistently originate a B cell precursor pre-leukemic stage. **a** Scheme of experimental cross, depicting genotype of progeny. Green (controls) and orange (IL-7R^mut^) animals were bled from week 4 and monitored up to week 104, unless disease ensued. A cohort of Cre-toxicity controls (blue) was also monitored that presented no disease. **b** TCRβ and CD19 fractions within CD45-positive cells in blood from control and IL-7R^mut^ animals. Each dot denotes an animal and mean value is shown. Two-tailed unpaired *t*-test. **c** CD19 fraction (left) and IgM versus IgD subpopulations within CD19 (right) in the blood of one representative animal from each group at 6 weeks of age. Numbers indicate frequencies of each quadrant or region. **d** CD93 and BP-1 expression within IgM^−^IgD^−^ cells in the blood from the same animals than in **c**. **e** Scatter plots summarizing data from all animals analyzed as in **c** and **d**. Ctrls: *n* = 8; IL-7R^mut^: *n* = 10. Each dot denotes an animal and mean value is shown. Two-tailed unpaired *t*-test. **f** Scatter plots showing fractions (top) and absolute numbers (bottom) in BM for the indicated populations in 4-week-old animals from Ctrl (*n* = 7) and IL-7R^mut^ (*n* = 4) animals. Two-tailed unpaired *t*-test. Source data are provided as a Source Data file.

Although some B-ALL cases harbor clonal *IL7R* gain-of-function mutations^15,16^, suggesting that IL7R activation can be an early event in leukemia development, there is no formal evidence that *IL7R* drives B-ALL. Moreover, studies addressing the leukemogenic potential of *IL7R* gain-of-function mutations relied on retroviral overexpression and subsequent transplantation into immunocompromised mice^20–22^. To more rigorously evaluate the capacity of mutant IL-7Rα to transform B cell precursors in vivo and to originate B-ALL, we first introduced a human type 1a mutant *IL7R* form^7,13^ into the mouse coding sequence and validated its ability to promote constitutive signaling and to transform Ba/F3 cells (Supplementary Fig. 1). Then, using a FLEx switch strategy, we generated conditional mutant *IL7R* knock-in mice (Supplementary Fig. 2) that we crossed with CD2-Cre animals (Fig. 1a) to produce progeny in which recombination occurs at the CLP stage^24^, allowing for physiological regulation of mutant *IL7R* expression in developing lymphoid cells. The preservation of the normal developmental patterns of IL-7Rα modulation in our model is best illustrated during thymic T cell differentiation, where IL-7Rα expression levels are tightly and dynamically regulated (Supplementary Fig. 3). Our strategy prevented mutant *IL7R* overexpression (Supplementary Fig. 4) and abnormal surface IL-7Rα upregulation (Supplementary Fig. 5), ensuring that putative functional impacts were strictly due to *IL7R* mutational activation.

As early as 4 weeks, *IL7R* mutant (CD2Cre^Pos^.Il7r^cpt/wt^) mice displayed decreased T cell and increased B cell frequency in the blood (Fig. 1b). Although T cell precursors expressed mutant *IL7R* transcript levels similar to those of B cell precursors (Supplementary Fig. 6) there was no major impact on T cell development in the thymus (Supplementary Fig. 7), or on the distribution of mature T lymphocyte subpopulations (Supplementary Fig. 8). Instead, *IL7R* mutation affected the B cell lineage leading to aberrant frequency of IgM^−^ IgD^−^ CD93^+^ BP-1^+^ B cell precursors in the blood (Fig. 1c–e) that resulted from a major expansion of B cell precursors in the bone marrow (BM) (Fig. 1f). This pre-leukemic phenotype, characterized by partial differentiation arrest and expansion of pro- and pre-B cells, affected all CD2Cre^Pos^.Il7r^cpt/wt^ mice. Pre-leukemic cells had higher viability, but not proliferation, than normal B cell precursors (Supplementary Fig. 9) and displayed enrichment in stemness genes and increased self-renewal potential, in some cases leading to leukemia development upon transfer into recipient mice (Supplementary Fig. 10).

### Mutant IL-7Rα drives B-ALL

**Fig. 2:**
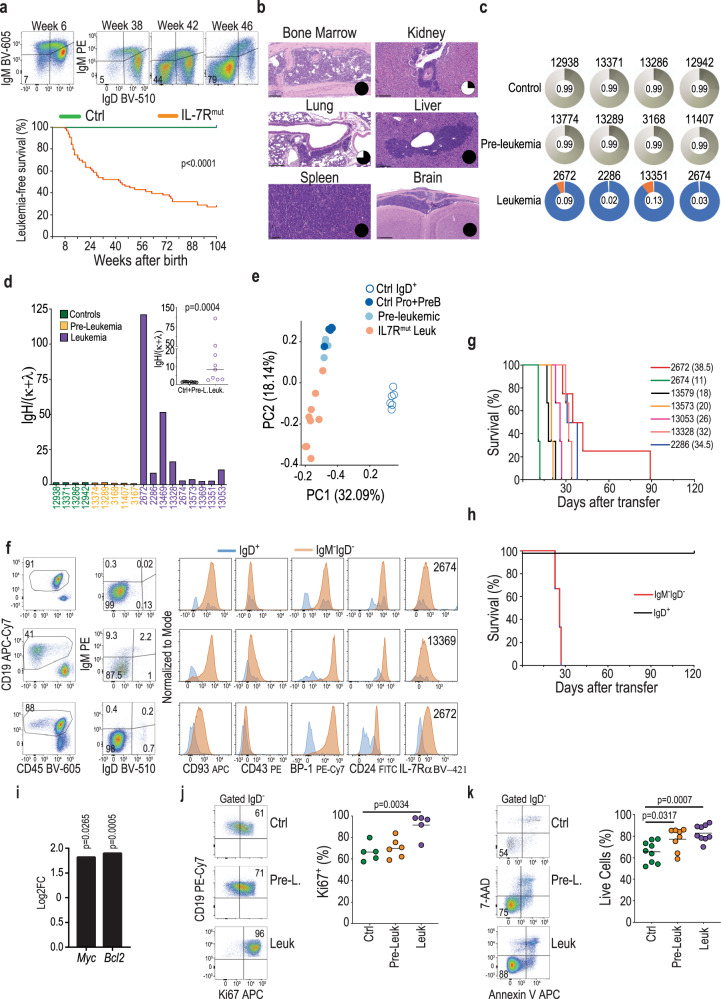
IL-7Rα mutant mice develop precursor B-ALL. **a** Example of IgM^−^IgD^−^ cell frequency evolution in a mouse that developed leukemia (top). Kaplan–Meier leukemia-free survival curves of control (Ctrl; *n* = 40) and *IL7R* mutant (IL7R^mut^; *n* = 63) animals (bottom). All mice died with precursor B-ALL. Log-rank Mantel–Cox test (**b**) Histologies (H&E) of organs infiltrated with leukemia cells. Pie chart inserts represent fraction of analyzed animals (*n* = 4) with leukemia involvement (in black) in the respective organ. Scale bar indicates 100 μm (bone marrow, spleen), 250 μm (kidney, lung, liver), or 500 μm (brain). **c** Clonality pie charts based on *IgH* sequencing. Each colored slice corresponds to a clone, indicative of clonality. Gray areas correspond to many rare clones, indicative of polyclonality. Equitability values (ranging from 0, for monoclonality, to 1, for a balanced repertoire) are shown in the center of the pie charts. Samples are bone marrow pro+pre-B cells from control, pre-leukemic, or leukemic mice. **d** Ig heavy chain (H) over light chain (κ and λ) ratios to evaluate, at the population level, the presence of the pro-B cell rearrangement signature (heavy chain expression in the absence of light chain expression). Two-tailed Mann–Whitney test performed. **e** Principal component analysis of normal (Ctrl Pro+Pre-B) and pre-leukemic (Pre-leukemic) pro- and pre-B cell precursors, mature splenic B cells (Ctrl IgD^+^) and leukemia cells (IL7R^mut^ Leuk). **f** Immunophenotypic analysis of three representative BM leukemia samples. Numbers inside dot plots indicate frequency in each quadrant or region. **g**, **h** Kaplan–Meier leukemia-free survival curves of mice transplanted with **g** bulk primary leukemias or **h** sorted IgD^+^ versus IgM^−^IgD^−^ leukemia cells. **i** *Myc* and *Bcl2* transcript upregulation (log2 fold change) in leukemia samples (*n* = 9) as compared to normal controls (*n* = 5). Moderated *t*-test performed. **j** Frequency of Ki67-positive cells in controls (*n* = 5), pre-leukemia (*n* = 5), and leukemia samples (*n* = 5). Dot plots are representative of each condition. **k** Frequency of annexin V/7AAD-negative (viable) cells in controls (*n* = 9) pre-leukemia (*n* = 8) and leukemia samples (*n* = 9). Dot plots are representative of each condition. **j**, **k** Two-tailed unpaired *t*-test. Source data are provided as a Source Data file.

**Fig. 3:**
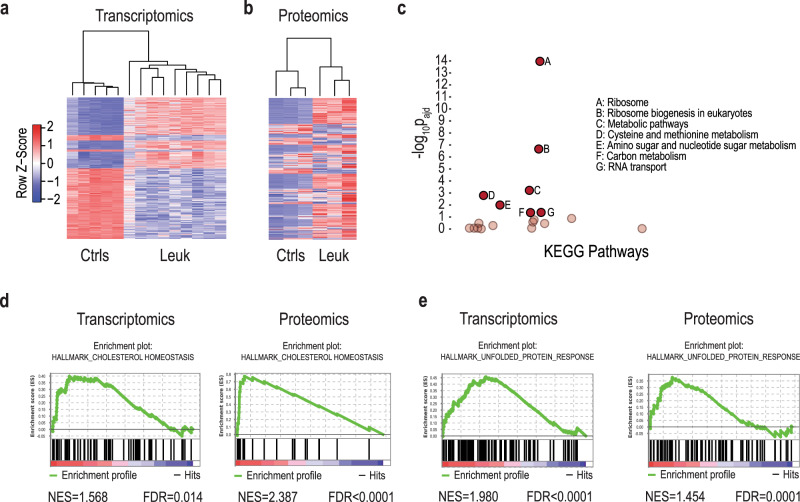
Transcriptomics and proteomics characterization of mutant *IL7R* leukemias. **a** Heatmap representation and hierarchical clustering of control and leukemia samples based on the 1000 most significant (adj. *p* value) differentially expressed genes. **b** Heatmap representation of samples based on the 500 most significant (nominal *p* value) differentially expressed proteins between control and leukemia samples. **c** g:Profiler KEGG pathway functional enrichment analysis for significant and concordantly upregulated genes and proteins in leukemia samples. Significantly enriched pathways (A–G, adj. *p* < 0.05. Cumulative hypergeometric test) are represented in full opacity. Pathways below the significance threshold are represented in low opacity. Pathways where *p* = 1 are not featured. **d**, **e** Gene set enrichment analysis (GSEA)-enrichment plot of differential gene and protein expression between leukemias and controls showing a significant upregulation of the **d** cholesterol homeostasis and the **e** unfolded protein response (UPR) hallmark gene sets (normalized enrichment score (NES) > 1, FDR < 0.05). Source data are provided as a Source Data file.

The pre-leukemic stage eventually evolved into overt fatal leukemia in a majority of the mice (Fig. 2a), with high penetrance (reaching > 60% at 80 weeks) and a median latency of 43 weeks and a range of 8 to >90 weeks. This broad range resembles that described in humans, where ALL patients of different ages (children, adolescents, and adults) can display *IL7R* mutations. In addition to the BM, leukemia cells spread to the spleen, kidney, liver, lung, and CNS (Fig. 2b). In contrast to control and pre-leukemic cells, leukemias were clonal, as assessed by analysis of *Ig* gene rearrangements (Fig. 2c and Supplementary Fig. 11), displayed a higher IgH/(κ + λ) ratio (Fig. 2d) than pre-leukemias and normal controls, and had a gene expression profile closer to that of pro- and pre-B cell precursors than of mature splenic B cells (Fig. 2e). In agreement, their maturation arrest ranged from early pro-B to the small pre-B cell stage (Fig. 2f and Supplementary Data 1), altogether indicating they corresponded to bona fide precursor B-ALL. In accordance, the V(D)J rearrangements of the major clone(s) in each leukemia were productive in some cases (e.g. #2672 and #13573, both arrested at the small pre-B cell stage) and non-productive in others (e.g. #2674, a pro-B cell leukemia; Supplementary Data 2). Leukemias were transplantable (Fig. 2g) and leukemia-propagating cells were IgM^−^IgD^−^ (Fig. 2h). As expected from IL-7R-driven tumors^25,26^, leukemic cells displayed high *Myc* and *Bcl2* levels (Fig. 2i), and higher proliferation (Fig. 2j) and viability (Fig. 2k) than normal and pre-leukemic B cell precursors. Moreover, they presented distinctive transcriptional (Fig. 3a and Supplementary Data 3–4) and protein (Fig. 3b and Supplementary Data 5 and 6) expression profiles, with enrichment for ribosome, oxidative phosphorylation, and spliceosome pathways (Supplementary Fig. 12). Transcriptome and proteome data integration confirmed the enrichment for ribosome biogenesis, as well as RNA transport and different metabolic pathways, including cysteine and methionine metabolism, amino sugar and nucleotide sugar metabolism, carbon metabolism (Fig. 3c), and cholesterol homeostasis (Fig. 3d). Moreover, we found an enrichment for unfolded protein response (Fig. 3e), which has been proposed as a valid therapeutic target in B-ALL^27^.

### Leukemic transformation of B cell precursors downstream from *IL7R* mutation associates with upregulation of IL-7R-mediated signaling activation

**Fig. 4:**
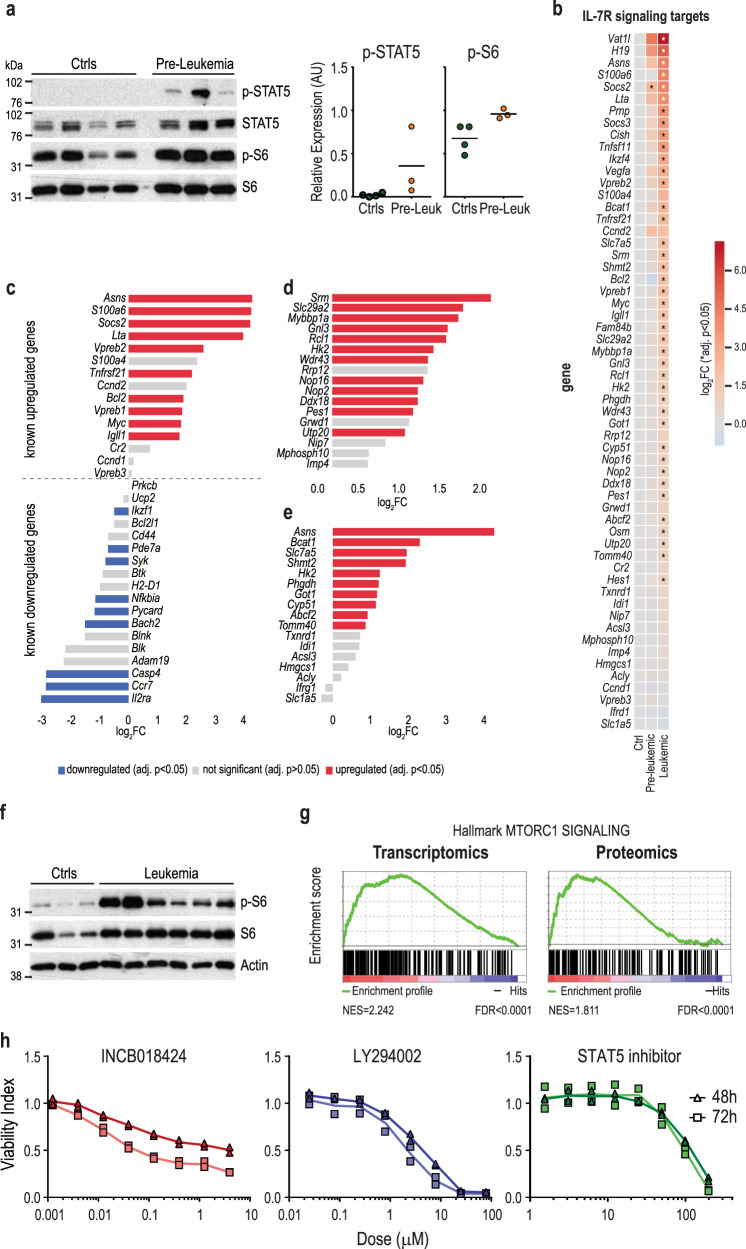
Leukemia cells rely on IL-7R signaling, which boosts upon transformation. **a** Immunoblot analysis of phosphorylated STAT5 and S6 levels in sorted pro+pre-B cells from control (*n* = 4) and pre-leukemic (*n* = 3) mice, IL-7-deprived for 12 h. Graphs represent densitometry values for P-STAT5 and P-S6 normalized to respective total protein. **b** Differential gene expression of known IL-7R signaling targets in pre-leukemia and leukemia samples compared to controls (reference category). Moderate *t*-test performed. **c**–**e** Differential expression of (**c**) STAT5, (**d**) MYC, and (**e**) mTOR targets in leukemia versus control samples. Significantly upregulated and downregulated genes (adj. *p* < 0.05; moderate *t*-test) are shown in red and in blue, respectively. **f** Immunoblot analysis of phosphorylated S6 levels in control (*n* = 3) and leukemia (*n* = 6) samples analyzed ex vivo. **g** Transcriptomic and proteomic gene set enrichment analysis (GSEA) showing a significant enrichment of the mTOR signaling hallmark gene set in leukemias versus controls. **h** Leukemia cells were cultured in the presence or absence of pharmacological inhibitors of JAK1/2 (INCB018424; ruxolitinib), STAT5 (STAT5 inhibitor), and PI3K (LY294002) at the indicated doses, and viability was evaluated at 48 and 72 h. Results from a representative mouse (*n* = 3). Lines represent average of duplicates that are shown. Source data are provided as a Source Data file.

We next sought to document IL-7R-mediated signaling alterations during the transformation process. Since healthy developing B lymphoid precursors are exposed to IL-7 in vivo, we cultured ex vivo pre-leukemic and normal B cell precursors in the absence of IL-7 to expose cell-intrinsic differences. We confirmed that pre-leukemia cells displayed cell-autonomous upregulation of IL-7R-mediated signaling, measured by phospho-STAT5 and phospho-S6 levels (Fig. 4a). In vivo, IL-7R signaling pathway target genes were mildly upregulated in pre-leukemia cells as compared to normal controls (Fig. 4b). However, leukemia establishment associated with marked IL-7R signaling upregulation (Fig. 4b). This was reflected in the activation of key downstream targets^26,28–31^, such as STAT5 (Fig. 4c), MYC (Fig. 4d and Supplementary Fig. 13), and mTOR (Fig. 4e–g). Accordingly, gene set enrichment analysis of pre-leukemic versus control samples showed several significantly upregulated pathways, including cholesterol homeostasis and the unfolded protein response, metabolism-related pathways (e.g. glycolysis, fatty acid metabolism, oxidative phosphorylation), and signaling pathways (MYC, PI3K/mTOR), which were further upregulated in leukemic mice (Supplementary Fig. 14). These observations align with IL-7R-mediated signaling increasing from control to pre-leukemia and then further augmenting upon conversion to leukemia. Importantly, leukemia cell viability relied on maintenance of high IL-7R-mediated signaling, as shown by inhibition of JAK1, STAT5, or PI3K/mTOR signaling (Fig. 4h), or by the use of the bromodomain inhibitor JQ1 to concomitantly target *IL7R* and *MYC* transcriptionally^32^ (Supplementary Fig. 15).

### Homozygous expression of mutant IL7R leads to early disease onset and full penetrance

**Fig. 5:**
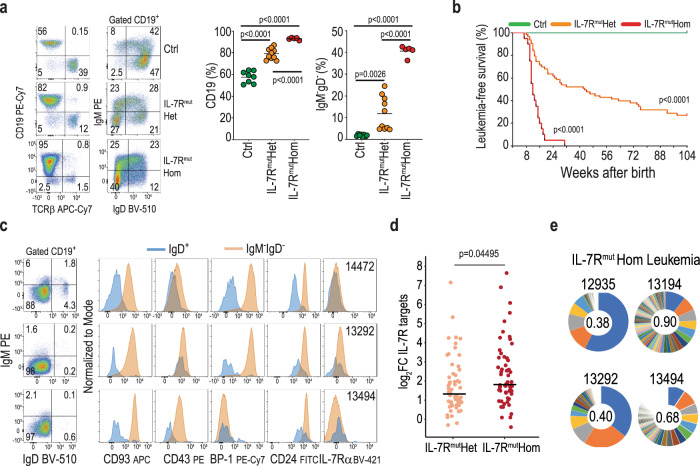
Homozygous expression of mutant IL7R leads to maximal IL-7R signaling hyperactivation and rapidly fatal leukemia. **a** Dot plots of CD19 by TCRβ to identify T and B cell lymphocytes in blood at 6 weeks of age in representative animals of indicated genotypes (left) and graphs summarizing data from all animals analyzed (right). Ctrls: *n* = 8; IL-7R^mut^Het: *n* = 10; IL-7R^mut^Hom: *n* = 5. Each dot denotes an animal and mean value is shown. Numbers in dot plots indicate frequency in each quadrant. One-way ANOVA. **b** Kaplan–Meier leukemia-free survival curves of control (*n* = 40), IL-7R^mut^Het (*n* = 63), and IL-7R^mut^Hom (*n* = 20) mutant animals. Log-rank (Mantel–Cox) test. All mice died with precursor B-ALL. **c** Immunophenotypic analysis of three representative BM IL-7R^mut^Hom leukemia samples. Numbers inside dot plots indicate frequency in each quadrant or region. **d** Comparison of IL-7R “signaling strength” between IL-7R^mut^Het and IL-7R^mut^Hom leukemia samples, as measured by the levels of differential gene expression over control samples. Each dot represents log2 fold change (FC) of an IL-7R signaling target gene (see Fig. 4b). Means are indicated. Unpaired two-tailed *t*-test. **e** Clonality pie charts based on *IgH* sequencing (see Fig. 2c for further details). Source data are provided as a Source Data file.

These observations suggested there was a strong selective advantage for B cell precursors to display high levels of IL-7R-mediated signaling on the path leading to leukemia development. If so, maximal IL-7R signaling should drive faster leukemia development. In agreement, homozygous expression of mutant *IL7R* in CD2Cre^Pos^.Il7r^cpt/cpt^ mice led to early accumulation of B cell precursors in the blood (Fig. 5a), rapidly followed by precursor B-ALL establishment (Fig. 5b, c, Supplementary Fig. 16 and Supplementary Data 1). Leukemia developed with very rapid kinetics (median latency of 12 weeks) and full penetrance at 31 weeks (Fig. 5b), and displayed highest IL-7R-mediated signaling levels (Fig. 5d and Supplementary Figs. 16 and 17). Leukemias from *IL7R* mutant homozygous mice were polyclonal (Fig. 5e) and tended to have less mutational burden, including of high and moderate impact variants (Supplementary Fig. 17) than those from *IL7R* heterozygous animals, suggesting that highest IL-7R signaling levels easily transform B cell precursors without requiring (a large number of) additional oncogenic hits. Overall, these results indicate that transformation downstream of *IL7R* mutational activation converges on the potentiation of IL-7R signaling itself and stronger activation overcomes more efficiently the barriers to B-ALL development.

### Mutant *IL7R* leads to the development of PAX5 P80R and Ph-like ALL

**Fig. 6:**
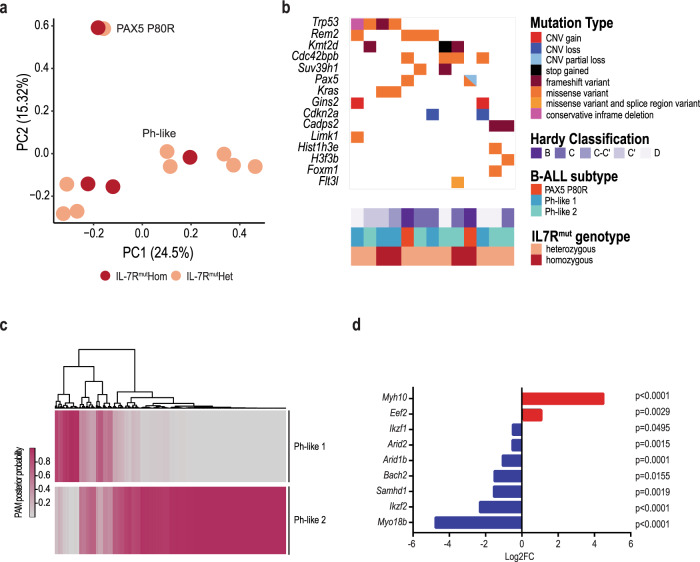
Mouse mutant *IL7R* leukemias resemble Ph-like and PAX5 P80R human B-ALL. **a** Principal component analysis (PCA) plot of gene expression profiles from mouse leukemia samples. **b** Mutational burden map of copy number variants (CNVs), single nucleotide variants (SNVs), and indel variants with predicted high and moderate impact in genes of interest. **c** Heatmap of PAM posterior probabilities classification of human Ph-like samples (from ref. ^16^) into the two apparent Ph-like subgroups observed in *IL7R* mutant leukemic mice. **d** Oncogenes and tumor suppressor genes significantly (adj. *p* < 0.05; moderate *t*-test) up- and downregulated, respectively, in leukemic samples as compared to controls. Log2 fold-changes (FC) are indicated for each gene. Source data are provided as a Source Data file.

A considerable fraction of human B-ALL cases from particular subtypes, especially PAX5 P80R and Ph-like, harbor *IL7R* mutations^16^. This raises the possibility that mutant *IL7R* may drive preferentially certain types of precursor B-ALL. In agreement, centroid analysis of the gene expression profile of *IL7R* mutant mouse tumors revealed they resemble precisely PAX5 P80R or Ph-like human B-ALL (Supplementary Data 7). Principal component analysis (PCA) confirmed the existence of two subgroups, which discriminated the cases identified as PAX5 P80R or Ph-like (Fig. 6a). Notably, whole-exome sequencing (WES) confirmed that the two mouse ALLs predicted to be PAX5 P80R had either *Pax5* P80R homozygous mutations or a heterozygous mutation and a deletion affecting the remaining allele (Fig. 6b and Supplementary Data 8 and 9). Sanger sequencing confirmed these results and identified 6 of 53 (11.3%) ALLs with PAX5 P80R mutations (Supplementary Figs. 18 and 19). These findings highlight the relevance of our model for human B-ALL. Further, similar to humans, Ph-like mouse ALLs displayed mutations in genes involved in signal transduction, such as *Kras*^33^, as well as *Flt3l, Limk1, Rem2* and *Cdc42bpb* (Fig. 6b and Supplementary Fig. 18). The latter two were also altered in one PAX5 P80R case. In addition, we found mutations in *Trp53*, *Kmt2d* and *FoxM1* (Fig. 6b and Supplementary Fig. 18), genes reported in human ALL^17,34,35^.

We noticed that PCA also segregated *IL7R* mutant-driven Ph-like ALLs into two transcriptionally distinct subsets (Fig. 6a), which we named Ph-like 1 and 2. The top 1000 genes that separated Ph-like 1 from Ph-like 2 in PC1 showed enrichment for KEGG antifolate resistance and JAK–STAT signaling pathways, whereas those related to Ph-like 2 were enriched in, for instance, Wnt and Hippo signaling pathways (Supplementary Data 10). In agreement, Ph-like 1 displayed a stronger profile of activation of IL-7R downstream target genes than Ph-like 2 (Supplementary Fig. 20). In accord with higher IL-7R-mediated signaling in Ph-like 1, we found transcriptional enrichment in MYC and mTOR signaling in the Ph-like 1 subgroup versus Ph-like 2 (Supplementary Fig. 20). The two subgroups associated with different mutational patterns, with Ph-like 2 presenting mutations in regulators of chromatin remodeling and transcription (*Kmt2d*, *Suv39h1*, *H3f3b* and *Hist1h3*) and exocytosis/secretion (*Cadps2*), that were absent from Ph-like 1 (Fig. 6b, Supplementary Fig. 21 and Supplementary Data 8). Conversely, *Cdkn2a* deletions, *Kras* mutations, and *Gins2* gains were present exclusively in Ph-like 1 (Fig. 6b, Supplementary Fig. 21 and Supplementary Data 8 and 9). Gins2 was reported to promote survival and proliferation of K562 cells and there is an association between Gins2 expression levels and response to MTX in ALL^36^. Of note, these two IL-7R-related subsets are present in human ALL cases (Fig. 6c and Supplementary Data 11).

Altogether, our data indicate that *IL7R* mutant-driven leukemias are either PAX5 P80R or Ph-like, in accord with these subtypes being enriched within mutant *IL7R* patients (Supplementary Data 12).

Transcriptional alterations in oncogenes and tumor suppressors may also potentiate or collaborate with the effects of *IL7R* mutation. In addition to *Myc* and *Bcl2* (Fig. 2i), other tumor-promoting genes such as *Eef2* and *Myh10* were upregulated in leukemia cells, whereas tumor suppressors such as *Ikzf1*, *Ikzf2*, *Arid1b*, *Arid2*, *Samhd1*, *Bach2*, and *Myo18b* were downregulated (Fig. 6d and Supplementary Data 4).

### *Kras* mutation cooperates with mutant *IL7R*

To provide evidence that the gene lesions we identified cooperate with *IL7R* mutation in driving B-ALL, we next transduced *IL7R* mutant BM progenitors with *Kras* Q61H and transplanted them into lethally irradiated mice (Supplementary Fig. 22A). *IL7R* mutant progenitors expressing Kras Q61H were able to reconstitute B cell development to a similar degree those transduce with empty vector or to *IL7R* wild-type progenitors expressing Kras Q61H (Supplementary Fig. 22B). However, *IL7R* mutant Kras Q61H-expressing B cell precursors (GFP+) displayed larger size (indicative of increased metabolism and proliferation), higher surface levels of the IL-7R downstream target ENPEP/BP-1, and higher levels of IL-7R signaling target genes *Cish*, *Asns*, and *Ccnd2*, as compared to control GFP-negative cells from the same transplants or empty vector-transduced cells (Supplementary Fig. 22C–E). *IL7R* wild-type precursors transduced with Kras Q61H confirmed that these differences resulted mostly from the cooperation between mutant *IL7R* and mutant *Kras*, rather than from the effect of each oncogene alone (Supplementary Fig. 22C–E). Importantly, one of seven transplanted IL7R mutant precursors transduced with Kras Q61H developed leukemia (Supplementary Fig. 23), whereas none of the controls showed any signs of leukemia development. These results indicate that *Kras* mutation cooperates with *IL7R* mutation in upregulating IL-7R-mediated signaling and accelerating leukemic transformation, suggesting that one of the possible mechanisms by which additional lesions cooperate with *IL7R* mutation in driving leukemia is the upregulation IL-7R-mediated signaling.

### Therapeutic targeting of *IL7R* mutant B-ALL

**Fig. 7:**
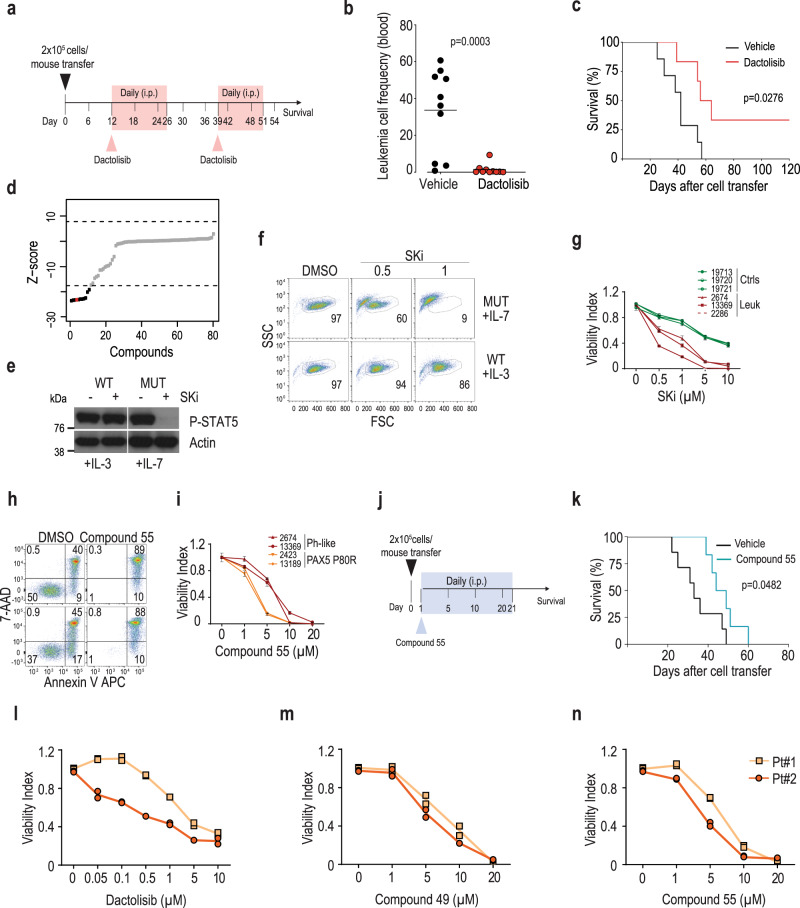
Mutant *IL7R* B-ALLs are sensitive to PI3K/mTOR and SK pharmacological inhibition. **a** Dactolisib in vivo administration scheme. Leukemic cells (2 × 10^5^) were transferred into Rag−/−γc−/− hosts. **b** Leukemia cell frequency detected in blood 6 days after treatment (*n* = 10). Two-tailed, unpaired *t*-test. **c** Kaplan–Meier survival curves, and respective log-rank *p* value, of animals (*n* = 7 control; *n* = 6 dactolisib) treated with vehicle or dactolisib (30 mg/kg). **d** *Z*-score values of chemical screen library for kinase inhibitors tested in IL-3-dependent Ba/F3 cells stably transduced with mutant *IL7R* and cultured in the absence of IL-3. Each compound is represented by one dot. SKi is denoted in red. Dotted lines represent *Z*-score cut-off. **e** Immunoblot analysis of STAT5 phosphorylation in Ba/F3 cells pre-treated with SKi (10 µM) or DMSO for 2 h and then stimulated with either IL-3 (*IL7R* WT cells) or IL-7 (*IL7R* mutant cells). *IL7R* WT-transduced cells were used merely as transduction controls for *IL7R* mutant cells but express IL-3R and respond to IL-3. IL-7 was used in IL7R mutant cells to reinforce IL-7R signaling while keeping the same period (30′) of cytokine stimulation as in IL-3-stimulated cells. This experiment was repeated once with identical results. **f** Viability, evaluated by FSC × SSC flow cytometry discrimination, of IL-3-cultured *IL7R* WT cells or IL-7-cultured *IL7R* mutant Ba/F3 cells treated with SKi or DMSO for 72 h. **g** Viability of leukemia cells from three independent mice, as compared to healthy B cell precursor controls in the presence of IL-7, cultured with increasing concentrations of SKi for 12 h. Viability index is normalized to the control condition (DMSO). Average ± s.e.m. is shown for each concentration. **h** Viability (Annexin V/7AAD expression) of representative leukemia cells incubated with SK2 inhibitor Compound 55 (20 µM) or DMSO for 12 h. **i** Dose-dependent effect of Compound 55 on leukemia cell viability (*n* = 4; 2 PAX5 P80R and 2 Ph-like). **j** Compound 55 in vivo administration scheme. Leukemic cells (2 × 10^5^) were transferred into Rag−/−γc−/− hosts. **k** Kaplan–Meier survival curves and respective log-rank *p* value of animals treated with vehicle (*n* = 7) or Compound 55 (*n* = 6; 5 mg/kg). **l**–**n** Viability of mutant *IL7R* human PAX5 P80R primary B-ALL cells cultured with increasing concentrations of (**l**) Dactolisib, (**m**) Compound 49, or (**n**) Compound 55 for 48 h. Viability index is normalized to the control condition (DMSO). Lines represent average of the duplicates, which are shown, for each patient sample. Source data are provided as a Source Data file.

We next explored our model to investigate ways to target therapeutically B-ALLs with IL-7R mutational activation. High PI3K/mTOR signaling pathway activation has been associated with disease aggressiveness and poor prognosis in B-ALL^37,38^. Because *IL7R* mutant leukemias exhibited clear upregulation of mTOR signaling, we administered the clinical-grade dual PI3K and mTOR inhibitor dactolisib (Fig. 7a) and found it had a striking impact on the frequency of leukemia cells in the blood (Fig. 7b), significantly prolonging the survival of transplanted mice (Fig. 7c). This is consistent with observations in human Ph-like ALL patient-derived xenografts using another PI3K–mTOR inhibitor^39^.

Next, to identify new targeted agents against mutant *IL7R* dependence, we performed a chemical screen using an 80-compound kinase inhibitor library on Ba/F3 cells ectopically expressing mutant IL-7Rα^13^. In addition to Cdk inhibitors, expected to counteract the effects of IL-7R-mediated signaling^40,41^, we found that a pan-sphingosine kinase (SK) inhibitor (SKi), which blocks the activity of both SK1 and SK2, efficiently prevented IL-7R-dependent cell growth (Fig. 7d and Supplementary Data 13). SK inactivation was not broadly cytotoxic, since the inhibitor abrogated only IL7R-, but not IL-3-, dependent signaling (Fig. 7e) and viability (Fig. 7f) of Ba/F3 cells. SKi also induced cell death in *IL7R* mutant mouse B-ALLs (Fig. 7g). We validated this effect using new generation SK inhibitors^42^. Both the dual SK1/SK2 inhibitor Compound 49 and the isoform-specific SK2 inhibitor Compound 55 (ref. ^42^) promoted dose-dependent leukemia cell death in vitro (Fig. 7h, i and Supplementary Fig. 24), and were effective in vivo (Fig. 7j, k and Supplementary Fig. 24).

Importantly, we validated our findings in human B-ALL. Similar to the mouse leukemia samples, pharmacological inhibition of PI3K and mTOR using dactolisib (Fig. 7l and Supplementary Fig. 25), or treatment with the SK inhibitors Compound 49 (Fig. 7m) and Compound 55 (Fig. 7n) had a clear inhibitory effect on two primary human diagnostic PAX5 P80R B-ALL samples presenting with *IL7R* type 1a gain-of-function mutations.

## Discussion

The IL-7/IL-7R signaling axis has been implicated in T- and B-ALL. However, the lack of evidence that *IL7R* mutational activation per se could trigger leukemogenesis and the absence of models where mutation could be uncoupled from overexpression of the receptor prompted us to develop a conditional mutant *IL7R* knock-in mouse. A study parallel to ours, published while our manuscript was under revision, made use of a conditional floxed transgenic model where the *IL7R* mutation was introduced into the Rosa26 locus and expressed in B cell committed precursors, and demonstrated that mutant *IL7R* can drive B-ALL^43^. However, the strategy used leads to the expression of the mutant receptor downstream of an ubiquitously expressed promoter, implicating that the mutant *IL7R* is not physiologically regulated (hence it will be either over- or ectopically expressed throughout B cell development) and is aberrantly expressed in the context of two wild-type *IL7R* alleles. This is of particular relevance, as we recently demonstrated that overexpression of the wild-type *IL7R* can be oncogenic^44^. In contrast, in our model, mutant *IL7R* expression is kept under physiological regulation—similar to what happens in human ALL, where no mutations in promoter, enhancer, or super-enhancer regions directly regulating *IL7R* have been reported. Our results demonstrate that *IL7R* gain-of-function mutation initiates leukemia by causing a pre-leukemic stage in B cell precursors with evidence of self-renewal activity. Our findings are coincident with those in human hematopoietic precursor cells, arising from parallel studies conducted by Geron et al., demonstrating the ability of mutant *IL7R* to induce a pre-leukemic stage with self-renewal ability (10.1101/2020.01.27.919951). We further show that pre-leukemia cells display higher viability, but not proliferation, than healthy B cell precursors and eventually transform, likely by the accumulation of mutations (e.g. Kras Q61H) that tend to contribute to increased IL-7R-mediated signaling and proliferation, into full-blown leukemia with characteristics of PAX5 P80R or Ph-like ALL. These are precisely the subsets where IL7R mutations are most frequent in humans^16,17^. The relatively long median latency for leukemia development in the *IL7R* mutant heterozygous mice is also consistent with the fact that Ph-like ALL is predominant in older patients. Although *IL7R* mutations are frequently subclonal in human ALL, indicating they occur late in leukemia development, there are cases of Ph-like B-ALL where *IL7R* mutant allele frequency is compatible with *IL7R* mutation being the initiating lesion^16,45^. Likewise, although *PAX5* P80R mutations are known to be the initiating event in this ALL subtype^16^, *IL7R* and *PAX5* mutant allele frequencies are, in some cases, compatible with the possibility of *IL7R* mutation initiating the disease with subsequent early acquisition of *PAX5* mutations^16^. In agreement, PAX5 P80R lesions occur in our IL-7R-driven model (in around 11% of the cases), suggesting a strong selective advantage in the combination of *IL7R* and *PAX5* mutations. Mutations and copy number alterations in genes affected in human B-ALL included also *Trp53*, *FoxM1*, *Cdkn2a*, *Kras*, and *Kmt2d*, underlining the relevance of our model to dissect the etiology of this malignancy. Moreover, lesions affecting these genes cooperate with *IL7R* mutation, likely contributing to increased proliferation, as shown by our studies with mutant *Kras* and consequent upregulation of *Ccnd2*, and those of Geron et al., which showed deletion of *CDKN2A* in a B-ALL arising from the in vivo transplantation of human cord blood progenitors transduced with mutant *IL7R* and demonstrated cooperation between *IL7R* mutation and *CDKN2A* deletion in driving human B-ALL (10.1101/2020.01.27.919951). Also noteworthy are the two IL7R-triggered Ph-like ALL transcriptional subgroups, one of which (Ph-like 1) displays *Cdkn2a* deletions, *Kras* mutations and *Gins2* gains and the other (Ph-like 2) mutations in genes encoding histone methyltransferases (*Kmt2d*, *Suv39h1*) and histone subunits (*H3f3b and Hist1h3*). Although kinase and signaling-related gene alterations, such as those affecting *Ras*, are a hallmark of human Ph-like ALL^46^, mutations in histones and chromatin regulators can also occur in this subset^17,47^. Notably, we found transcriptional counterparts to both mouse Ph-like subsets in human leukemia. Nonetheless, the two Ph-like transcriptional subsets we identified and their respective mutational characteristics need further validation in a larger number of animals and, especially, in the human setting.

In our model, *IL7R* mutational activation occurred from the CLP stage onwards. However, we did not detect any major alterations in T cell development or the occurrence of T cell ALL, despite obvious expression of the mutant *IL7R* in the T cell lineage. In T cells, IL-7R-mediated signaling leads to *IL7R* transcriptional downregulation^48^, and IL-7 stimulation decreases IL-7Rα surface expression due to increased trafficking to the early endosome and subsequent degradation^49^. These constitute obstacles to excessive signaling in T cells not shared by B cell precursors, which may help explain the skew towards B-ALL that we observed. The limits to IL-7R-mediated transformation in B- versus T-cell precursors warrant further investigation.

Leukemia development occurs in a majority of heterozygous, and in all homozygous, *Il7r* mutant mice, contrasting with previous studies showing that STAT5 activation alone leads to B-ALL with very low penetrance and long latency^50^. This indicates that IL-7R-mediated leukemogenic effects extend beyond those of the JAK–STAT pathway, in line with the knowledge that IL-7R triggers other oncogenic signaling pathways^7^. In fact, we showed that *IL7R* mutant ALL cells rely not only on JAK and STAT5, but also on PI3K–mTOR activity. These observations suggest that the combination of distinct survival and proliferative pathways (e.g. JAK–STAT and PI3K–mTOR) triggered by mutant *IL7R* likely compensates for possible barriers to transformation imposed to the activation of each pathway separately^51^, overall resulting in strong oncogenic potential. The ability of high IL-7R-mediated signaling levels to drive ALL is illustrated in mutant *IL7R* homozygous mice, whose B cell precursors undergo transformation in a very rapid and polyclonal fashion (indicating a strong ability to transform), and with less moderate/high impact mutations (indicating higher signaling requires less cooperating hits to drive leukemia). The results from *IL7R* homozygous mice would also be compatible with wild-type IL-7R acting as a tumor suppressor. However, this is unlikely, since most evidence in T- and B-ALL suggests that high IL-7R wild-type signaling is oncogenic^11,25,44,52^. While increased signaling in homozygous versus heterozygous *IL7R* mutant leukemias may potentially result from increased expression of the mutant form and/or absence of the wild-type IL7R, the end-result is markedly increased signaling and increased oncogenic potential.

We identified transcriptional and mutational alterations that may contribute to leukemogenesis downstream from *IL7R* mutation. IL-7R-dependent *Myc* upregulation is an obvious candidate, especially taking into account that survival effectors (such as Bcl-2) activated by mutant IL-7R-mediated signaling will likely counterbalance possible pro-apoptotic effects of Myc activation^53^. *MYH10* encodes non-muscle myosin heavy chain IIb, and is upregulated in different cancers, including myeloid leukemias^54^. *Myh10* was upregulated in *IL7R* mutant leukemias. Curiously, other regulators of motility were also affected in the leukemic samples, namely the tumor suppressor *Myo18b*^55^ that was downregulated, and *Cdc42bpb* (encoding Mrckb) and *Limk1* (known to be phosphorylated by Mrckb) that were mutated. Altogether, this suggests that IL-7R signaling can activate and/or cooperate with a program involved in tumor spreading. Whereas we did not find mutations in tumor suppressors such as *Arid1b*, which is mutated in human B-ALL^17^, there was clear transcriptional downregulation that extended to *Arid2*. Similarly, we did not detect genetic alterations in *Ikzf1* (encoding Ikaros), and found only mild downregulation in *Ikzf1* transcript levels. Although *IKZF1* deletions are a hallmark of Ph-like ALL^15,56^, they in fact tend not to co-segregate with *IL7R* mutations within this subset. Only 20% of exon 6 *IL7R* indel mutant cases display *IKZF1* alterations, whereas samples with ABL-class fusions (71%), CRLF2 rearrangements (77%), and EPOR or JAK2 rearrangements (86%) show considerably higher frequencies^15^. Mechanistically this is not surprising, since Ikaros competes with IL-7R-dependent signaling in B cell development and is displaced by STAT5 during leukemogenesis^50,57^. This implies that high signaling levels from mutant IL-7Rα will tend to render Ikaros functionally obsolete without the need for mutational inactivation.

Comparison of drug sensitivity of mutant *IL7R* human B-ALL samples with B-ALLs arising in our mouse model validated its use in the search for novel therapeutic avenues. Previous studies showed that BET bromodomain^32^ or JAK^58^ inhibitors can target IL-7R-related high risk ALL. Here, we demonstrated that PI3K-mTOR inhibition is an additional strategy—of particular relevance given the association of mTOR activation with poor prognosis in B-ALL^37,38^. Moreover, in the sequence of our kinase-directed functional screening, we demonstrated that SK pharmacological inhibitors abrogate IL-7R-mediated signaling, promote mutant *IL7R* ALL cell death, and delay tumor growth. This is in line with the knowledge that SK activity promotes B-ALL^59^, and indicates that SK small-molecule inhibitors may be particularly useful against IL-7R-dependent B-ALL cases.

In summary, we generated an in vivo model that formally demonstrates that *IL7R* gain-of-function drives bona fide precursor B-ALL and, importantly, can initiate the disease. Our model constitutes a resource to characterize new molecular and cellular players in B-ALL etiology, in the context of otherwise normal hematopoietic development, and to test new therapeutic approaches in an immunocompetent setting.

## Methods

### Mouse models

All animal experiments were performed according to the regulations of Instituto de Medicina Molecular João Lobo Antunes (IMM-JLA) and Portuguese and European legislation. *Il7r*^*flCPT*^ conditional knock-in animals in C57Bl/6 background were generated by Cyagen Biosciences (Santa Clara, CA). *Il7r* exon 6 was targeted by a homologous recombination vector carrying the wild-type exon 6 and a reversely positioned mutant exon 6 in conjunction with a Neomycin (Neo) selection cassette and flanking 5′ and 3′ homology arms. The mutant exon 6 consisted of an in-frame insertion of TGTCCCACC, coding for cysteine (C), proline (P), and threonine (T), at the coding sequence nucleotide position 733 (Supplementary Fig. 1A). The Neo cassette, flanked by Frt sites, was deleted from the conditional knock-in (KI) allele by crossing the transgenic mice with a strain expressing the yeast Flp recombinase. Supplementary Figure 2A shows the conditional knock-in allele after Neo deletion. Exon 6 and mutant exon 6 were flanked by LoxP and Lox511 sequences in a FLEX switch configuration^60,61^. The LoxP and the variant Lox511 sites were arranged in such a configuration (place, distance, and orientation) that, upon Cre recombinase activity, the mutant and wild-type exons 6 are inverted, positioning the mutant exon 6 in the sense orientation and deleting the wild-type exon 6 (Supplementary Fig. 2A). In keeping with the guidelines set by the International Committee on Standardized Genetic Nomenclature for Mice, our KI mouse strain was named C57BL/6J-Il7r <tm1(CPT)> or B6-Il7rtm1(CPT) where “(CPT)” accounts for the CysProThr insertional-mutation. Hereafter, it will be referred to as *Il7r*^*flCPT*^ (fl = floxed). Genotyping of the *Il7r*^*flCPT*^ allele was carried out with primers F1 and R1 (all primer sequences can be found in Supplementary Data 17). Heterozygous mutant mice were identified through the presence of a 352 bp fragment from the wild-type allele and of a 521 bp fragment resulting from the recombinant allele. To confirm the Cre-mediated recombination of the floxed *Il7r*^*flCPT*^ allele, primer F1 and F2 were used. This results in a 1067 bp band from the loxP/Lox511 inverted/excised allele. hCD2-iCre, B6 Rag^−/−^ γc^−^^/−^ and NSG animals were bred and kept at the IMM-JLA SPF animal facility. In all experiments, animals were closely monitored and sacrificed when reaching humane endpoints: loss of 20% of body weight, hind leg paralysis, breathing impairment, or poor reaction to external stimuli. Experiments were performed according to the IMM-JLA’s institutional (including ORBEA Ethical Review approval) and Portuguese (DGAV) regulations. Animals were kept in ventilated systems at 20–24 °C, and relative humidity of 55 ± 10%, with controlled supply of High Efficiency-Particulate Air (HEPA) filtered air provided to individually ventilated cages. Maximum number of animals per cage was 5. Social isolation was avoided whenever possible. Rooms’ light/dark cycle was 14 h Light:10 h Dark. Type of food was autoclaved diet pellets RM3A (P), from SDS Special Diets Services (Product code: 801030). Food was placed in a grid inside the cage and provided ad libitum to animals.

### Leukemia incidence

A cohort of experimental animals was weekly weighed and bled, and sacrificed in a CO_2_ chamber or via pentobarbital injection when reaching humane endpoints. In addition to the BM, disseminated disease was confirmed by collecting thymus, spleen, and liver samples for flow cytometry and histological analysis. Experiments were terminated at 104 weeks of age. Differences in survival curves were determined by Log-rank (Mantel–Cox) test using Prism v8.0.

### Leukemic cell transfer

2 × 10^5^ sorted leukemic cells were transferred i.v. via the tail vein into sex- and age-matched 8–20-week-old hosts. Animals were monitored daily, weighed, and bled for immunophenotyping weekly and sacrificed in a CO_2_ chamber or via pentobarbital injection when reaching humane endpoints. In the absence of disease, experiments were terminated 120 days after transfer.

### Self-renewal experiments

8–10-week-old sex-matched hosts were sub-lethally irradiated (350 rad) and transferred with 2 × 10^6^ sorted pro+pre-B cells from control or IL-7R^mut^ animals in first transfer and 10^7^ total BM cells in secondary transfers. Animals were monitored daily, weighed weekly, and sacrificed in a CO_2_ chamber or via pentobarbital injection when reaching humane endpoints.

### In vivo Ba/F3 tumor growth

Ba/F3 cells expressing the murine wild type or mutant *Il7r* were injected subcutaneously into the right and the left flank (respectively) of 11-week-old NSG mice (5 × 10^6^ cells in 100 μl of PBS). Mice were monitored for tumor development and tumor size measured by a caliper. Tumor volume was calculated using the formula: tumor volume = ½ (length × width^2^). Mice were euthanized when tumors reached a volume of 1000 mm^3^.

### In vivo treatment of leukemia

Sorted leukemia cells (2 × 10^5^) were transferred into Rag^−/−^γc^−/−^ hosts and animals were monitored daily and minimally bled (<20 µL) for FACS determination of leukemia cells (CD19^+^) at days 7 and 10 after transfer. Upon leukemia detection in all animals (mCD45^+^CD19^+^ > 0.5%), mice were randomized into treated and control groups. The PI3K/mTOR inhibitor Dactolisib (BEZ235, NVP-BEZ235; Selleckchem)—dissolved v/v 10 NMP and 90 PEG300 (Sigma Aldrich)—was administered daily at 30 mg/kg by oral gavage for 15 days, whereas vehicle was administered to the control group. Treatment was then stopped for 12 days and subsequently restarted for 13 days. Animals were sacrificed when reaching humane endpoints. Animals without disease symptoms were sacrificed 120 days after cell transfer. For sphingosine kinase inhibitor treatment, sorted leukemia cells (2 × 10^5^) were transferred into Rag^−/−^γc^−/−^ hosts and 1 day after injection animals were randomized into three groups: compound 49, compound 55, or vehicle (20% cyclodextrin/PBS). Both compounds were administered daily at 5 mg/kg by intraperitoneal injection for 21 days. Animals were monitored daily and bled weekly for FACS detection of leukemia cells (CD19^+^). Animals were sacrificed when humane endpoint was reached. Differences in survival curves were determined by log-rank (Mantel–Cox) test using Prism v8.0.

### Histopathology

Samples were immersion-fixed in 10% neutral buffered formalin, routinely processed for paraffin embedding, sectioned at 4 µm, stained with hematoxylin and eosin, and examined by a pathologist blinded to experimental groups. Tumor cell infiltration was scored according to a 5-tier severity scale: 0, absent; 1, minimal; 2, mild; 3, moderate; 4, marked, and representative photographs were acquired using NDP.view2 software (Hamamatsu) in slides digitally scanned in the Hamamatsu NanoZoomerSQ.

### Immunophenotyping

Blood was collected into tubes with heparin and red blood cells (RBCs) were lysed with lysis solution (Becton Dickinson San Jose, CA, USA), prior to staining with standard procedures. Splenic, thymic and BM single-cell suspensions were immunophenotyped using standard methodology. Briefly, 10^6^ cells were stained for 20 min at 4 °C in PBS with 2% FBS with specific antibodies (Supplementary Data 14). When lineage-positive cells were excluded, biotin coupled anti-Gr-1, CD11b, CD19, Ter119, NK1.1, and CD11c were used and subsequently stained with BV711 streptavidin. Proliferation was analyzed by intracellular staining of Ki67 (APC-conjugated, Biolegend), using the Foxp3 staining kit from eBioscience and following the manufacturer’s instructions. Cell viability was determined using an annexin V-based apoptosis detection kit and following the manufacturer’s instructions (eBioscience). 10-color analyses were performed on LSR Fortessa II (Becton Dickinson San Jose, CA, USA) flow cytometers. Results were analyzed with FlowJo (Tree StarInc., Ashland, OR, USA) software. Gating strategies used are exemplified in Supplementary Figs. 26–30.

### FACS cell sorting

BM and spleen cell suspensions were stained for sorting with anti-CD19, B220, IgM, IgD, CD45, and TCRβ and sorted on a FACSAriaIII (Becton Dickinson San Jose, CA, USA). Leukemia cells were sorted from BM or spleen samples as CD45^+or^^−^CD19^+^IgM^−^IgD^−^; pro+pre-B precursors were sorted from the BM as CD45^int^B220^int^IgM^−^; mature B cells were sorted from the spleen as CD45^+^CD19^+^IgD^+^; and T cells as CD45^+^TCRβ^+^. Retrovirally transduced cells were stained and sorted as CD19^+^GFP^+^ or CD19^+^GFP^−^, 3 days after transduction. Cells were then resuspended in IMDM for further culture or in PBS for transfer into recipient animals.

### WES and data analysis

DNA from sorted cells was extracted with Qiagen ALLprep extraction kit. Sequencing was performed as previously detailed^16^. WES data processing and variant calling were performed by RubioSeq software v3.8a (http://rubioseq.bioinfo.cnio.es/) using default parameters for somatic variation analysis^62^. Briefly, sequencing data were first checked by FastQC for quality control on raw sequence data and then aligned to the mouse reference genome (mm10) using Burrows-Wheeler alignment (BWA)^63^. Reads unmapped by BWA were realigned using BFAST^64^. For variant calling we used GATK Unified Genotyper v2 (ref. ^65^), applying the “Discovery” genotyping mode and default parameters for filtering. The GATK QUAL field was employed for ranking selected somatic variants. Variants were annotated with SnpEff 4.3r (VEP)^66^. Variants were filtered to ensure that each variant had at least a coverage over 30. Additional filters were applied for the specific identification of somatic variants; *loci* showing high numbers of variants exclusively present in sibling mice were not considered, and variants present in the Mouse Genome Project^67^ most recent release (v6) for SNPs and indels were also filtered out. For somatic copy number variation calling, the aligned WES bam files of leukemic and control mouse models as well as the corresponding bed file indicating exon target positions were used for somatic copy number variation (CNV) calling using the CODEX2 pipeline^68^. As a case–control design, CNVs disproportionately present in the leukemic mouse models compared to the controls were detected. In brief, GC content and mappability for each exon region was calculated from the mm10 reference genome. Depth of coverage matrices for each chromosome was built and sample- and exon-wise quality control regarding coverage, length, mappability, and GC content, was performed using default settings, before subjected to the CODEX2 algorithm background estimation, normalization, and CNV detection based on poisson-likelihood based recursive segmentation^69^. The fractional mode was used as the objective is to call somatic variant. The CNV events were categorized and number-coded to be amplification (+2), gain (+1), diploid (0), heterozygous deletion (−1), and homozygous deletion (−2), for cases where the estimated exact copy number ratio was >3.3, 2.3–3.3, 1.7–2.3, 0.7–1.7, and <0.7, respectively, as previously described^68^. The categorical CNV events were then visualized and examined using the Integrative Genomics Viewer version 2.4.16 (ref. ^70^). Waterfall plot for variants was generated using R (v3.4.4, https://www.R-project.org/) and the GenVisR package (v1.8.1).

### RNA sequencing (RNA-seq) and data analysis

RNA was extracted from sorted cells with Qiagen ALLprep extraction kit. Sequencing was performed as previously described^16^. RNA-seq data quality was assessed using FASTQC (v0.11.7, https://www.bioinformatics.babraham.ac.uk/projects/fastqc/). Reads were pseudo-aligned to mouse transcriptome (gencode M14) with Kallisto v0.44.0^71^. Differential expression was assessed using edgeR (v3.20.9) and limma (v3.34.9) R packages^72,73^. Briefly, samples comparison was performed using voom transformed values, linear modeling and moderated *T*-test as implemented in limma R package, selecting significantly differentially expressed genes with FDR adjusted *P* values lower than 0.05. *IL7R* allele-specific expression levels were obtained using samtools^74^ and bedtools^75^, converting to allele proportions for reference (wild-type) and mutant IL7R alleles. Principal component analysis (PCA) was performed using the stats package (v3.4.4) on voom transformed expression values. PCA plots were generated using the ggfortify (v0.4.8) and ggplot2 (v3.2.1) packages in R. Heatmap of transcriptional differences was generated with R package gplots with clustering performed with hclust from the R stats package. IL-7R, STAT5, Myc, and mTOR signaling targets were curated from the literature, differential expression heatmaps were generated with the seaborn (v0.9.0) and matplolib (v3.1.0) packages in Python 3.7.3, barplots were generated with ggplot2. We used MixCR framework to quantify clonotypes from transcriptome profiles using the recommended protocol for RNA-Seq^76^. Classification of IL-7R mutant mouse tumors was performed using human B-ALL transcriptome profiles^16^ and nearest shrunken centroids as implemented in pamR (v1.56.1) R package (https://cran.r-project.org/web/packages/pamr/index.html). Briefly, human-mouse orthologs were first obtained from Ensembl v95 through “biomaRt” R package^77^. Second, human and mouse transcriptome profiles were quantile normalized using preprocessCore (v1.40.0) R package (https://github.com/bmbolstad/preprocessCore). Third, the human transcriptome data were split in two sets enclosing seven human B-ALL subtypes known to display (PAX5 P80R, IKZF1 N159Y, iAMP21, Ph-like) or not (DUX4, ETV6-RUNX1, and KMT2A) *IL7R* mutations: approximately 74% of samples within each subtype were used to train the classifier and 26% to evaluate its prediction capability. Finally, the classifier was used to assign the *IL7R* mutant mouse tumors according to the human B-ALL subtypes. The pamR packaged was also used to assess the distribution of human Ph-like B-ALL samples^16^ among the seemingly two subgroups of Ph-like mice leukemias identified, where all mice from both subgroups were utilized for training of the classifier.

### Sanger sequencing

Genomic DNA from sorted leukemia cells was extracted using AllPrep® DNA/RNA/Protein kit (Qiagen) following the manufacturer’s instructions. PCR was performed with 50 ng of genomic DNA using either *Taq* DNA Polymerase (Thermo Scientific) or Recombinant Taq DNA Polymerase TaKaRa Taq™ (Takara Bio) using the primers listed in Supplementary Data 15. Sanger sequencing was performed using GATC services.

### Quantitative real-time PCR

RNA was retro-transcribed using High Capacity RNA-to-cDNA Kit, followed by a pre-amplification PCR using TaqMan PreAmp Master Mix. TaqMan Gene Expression Master Mix was used in real-time quantitative PCR performed in a 7500fast or ViiA7 real-time system (all from Applied Biosystems). TaqMan Gene Expression Assay (Applied Biosystems) was *Hprt1* (Assay ID: Mm00446968_m1), *Asns* (Assay ID: Mm00803785_m1), *Ccnd2* (Assay ID: Mm00438070_m1), and *Cish* (Assay ID: Mm01230623_g1). Expression of the mutated form of *Il7r* was detected in cDNA obtained from leukemic samples by RT-PCR analysis using custom Taqman probes and Gene Expression Master Mix. Expression of the mutant form of *Kras* (Q61H) was detected in retrovirally transduced cells, as described above, using Syber Green dye according to the manufacturer’s instructions. Gene expression analysis from RT-PCR analysis was calculated with the comparative CT method 2^−ΔΔCT^.

### FASP processing of samples for proteomics

Cell pellets were solubilized in Tris-HCl (100 mM, pH 8) containing 4% SDS and 100 mM DTT. Cell lysates were heated at 95 °C, and DNA was shredded by sonication. Samples were processed using FASP protocol^78^ with some modifications. After, lysates were passed through filters (Nanosep, 10k, PALL Life Sciences), proteins were alkylated in 100 µL iodoacetamide at a final concentration of 50 mM for 15 min, filters were washed three times with 200 µL 8 M urea in Tris-HCl (100 mM, pH 8) then twice with 200 µL 40 mM ammonium bicarbonate. Proteins on the filters were then digested twice at 30 °C with trypsin (3.3 µg ×2), first overnight and then for another 6 h in 200 µL, ammonium bicarbonate at 40 mM. Resulting tryptic peptides were desalted using C18 solid phase extraction cartridge (Empore, Agilent technologies).

### LC-MS analysis for proteomics

Analysis of peptides was performed on a Q-exactive-HF-X (Thermo Scientific) mass spectrometer coupled with a Dionex Ultimate 3000 RS (Thermo Scientific). LC buffers were the following: buffer A (0.1% formic acid in Milli-Q water (v/v)) and buffer B (80% acetonitrile and 0.08% formic acid in Milli-Q water (v/v). Aliquots of 5 μL of each sample were loaded at 10 μL/min onto a trap column (100 μm × 2 cm, PepMap nanoViper C18 column, 5μm, 100 Å, Thermo Scientific) equilibrated in 2% buffer B. The trap column was washed for 4 min at the same flow rate and then the trap column was switched in-line with a Thermo Scientific, resolving C18 column. The peptides were eluted from the column at a constant flow rate of 300 nL/min with a linear gradient from 5% buffer B to 35% buffer B in 116 min, and then to 98% buffer B by 118 min. The column was then washed with 98% buffer B for 15 min and re-equilibrated in 2% buffer B for 31 min. Q-exactive HF-X was used in data-dependent mode. A scan cycle comprised MS1 scan (*m*/*z* range from 335 to 1800, with a maximum ion injection time of 50 ms, a resolution of 60,000 and automatic gain control (AGC) value of 3 × 10^6^) followed by 40 sequential dependent MS2 scans (with an isolation window set to 1.4 Da, resolution at 7500, maximum ion injection time at 50 ms and AGC 10^5^, stepped collision energy was set to 27, and fixed first mass to 120*m*/*z*. Spectrum was acquired in centroid mode and all unassigned charge states as well as singly charged species were rejected. To ensure mass accuracy, the mass spectrometer was calibrated on the first day that the runs were performed.

### Proteomics data analysis

Raw mass spec data files were searched using the MaxQuant software package (version 1.6.0.1). Proteins and peptides were identified using a Uniprot canonical plus isoforms database (accessed 02/08/2017). The following search parameters within MaxQuant were selected: protein N-terminal acetylation and methionine oxidation were set as variable modifications and carbamidomethylation of cysteine residues was selected as a fixed modification; trypsin selected as digestion enzyme with up to two missed cleavages; the false discovery rate was set at 1% for protein and peptide and the match between runs function was disabled. Proteins were removed from the data set which were categorized as “reverse”, “contaminant” or “only identified by site”. Estimates of protein copy numbers per cell were calculated using the histone ruler method^79^ within the Perseus package^80^. Differential protein expression was performed using the DEP (version 1.0.1) and limma (v3.34.9) packages in R (v3.4.4). Molecules showing nominal *p* < 0.05 were considered significant.

### Pathway enrichment analysis

Two approaches were used to perform pathway enrichment analysis. Gene set enrichment analysis (GSEA) was performed on transcriptomic and proteomic data with GSEA v.4.0.3 (ref. ^81^), using the GSEAPreranked method with limma moderate t-statistic values as the gene ranking statistic against Molecular Signature Database (MsigDB)’s hallmark gene sets^82^ using 1000 permutations with a specific seed for reproducibility. Pathways with a false discovery rate (FDR) < 0.05 were considered significant. Additionally, a more integrative approach was taken by identifying concordantly significantly differentially expressed genes (adj. *p* < 0.05) and proteins (nominal *p* < 0.05) between IL-7R^mut^het and controls. Genes in these conditions were queried in g:profiler’s g:GOSt functional profiling tool against Kyoto Encyclopedia of Genes and Genomes (KEGG) pathways. Significance was set at adj. *p* < 0.05 using the tool’s suggested g:SCS multiple comparison correction method. Enrichment plots were generated automatically by the GSEA software, while custom plots were generated using the ggplot2 R package.

### Immunoblotting

Cell lysates were resolved by 12% SDS-PAGE and equal amounts of protein were transferred onto nitrocellulose membranes, and immunoblotted with antibodies against: p-STAT5 (Y694), p-S6(S235/236), p-Akt (S473), STAT5, S6 and Akt (Cell Signaling Technology), and β-actin (Santa Cruz Biotechnologies), as indicated in Supplementary Data 16. Immunodetection was performed by incubation with horseradish peroxidase-conjugated secondary antibodies (Promega Corporation) and developed by chemiluminescence (Thermo Scientific; GE HealthCare).

### In vitro drug testing

Leukemia cells were thawed and live cells magnetically sorted, according to the manufacturers’ instructions and specific alterations: we used an APC-conjugated Annexin V (invitrogen) to mark early/late apoptotic cells followed by secondary marking with anti-APC magnetic beads (Miltenyi Biotec), using Annexin V Binding Buffer (PromoKine). Cells were then cultured in duplicates as 10^6^ cells/mL in IMDM GlutaMAX™ medium (supplemented with 10% FBS, 1% Pen/Strep, 1% MEM, 1% sodium pyruvate, 0.1% gentamicin, 0.1% β-mercaptoethanol) for the indicated time points in the presence or absence of the indicated concentrations of the Jak1/2 inhibitor INCB018424 (Selleckchem), the PI3K inhibitor LY294002 (Merck-Calbiochem), or the STAT5 small-molecule inhibitor Nʹ-((4-Oxo-4H-chromen-3-yl)methylene)nicotinohydrazide (Merck-Calbiochem). These inhibitors were added right after plating and dispensed in eight dilution steps using the D300(e) Digital Dispenser (Tecan). DMSO concentration was normalized to 0.4% in each well. Dilutions were done following a logarithmic distribution (INCB018424 and LY294002) or linear distribution (STAT5 Inhibitor). Cell viability was assessed at the indicated time points by flow cytometry analysis of forward scatter (FSC) versus side scatter (SSC) discrimination. The pharmacological inhibitors Sphingosine Kinase inhibitor (SKi) (Calbiochem), and compounds 49 and 55 (kindly synthesized by David R. Adams), the PI3K/mTOR inhibitor Dactolisib (BEZ235, NVP-BEZ235; Selleckchem), and the BET bromodomain inhibitor JQ-1 (Sigma Aldrich) were used to evaluate impact on cell viability. IL7R^mut^ leukemia cells were thawed, and viable cells were either density-centrifuged using ficoll-paque or selected by Annexin V magnetic-activated cell sorting as described above. Cells were plated in a round-bottom 96-well plate as 10^6^ cells/mL and incubated with increasing concentrations of sphingosine kinase pharmacological inhibitors or vehicle (DMSO). Human B-ALL diagnostic samples with *IL7R* type 1a mutations were previously collected from adult patients, at Saint-Louis Hospital, Paris, after informed consent. Patients were enrolled in GRAALL-2014 clinical trial (NCT02617004), approved by the French Comité de protection des personnes (CPP) and the Agence nationale de sécurité du médicament et des produits de santé (ANSM). BM mononuclear cells were isolated by Ficoll-paque gradient centrifugation and cryopreserved in liquid nitrogen. Thawed cells were treated as described above for mouse leukemia cells. For *IL7R* wild-type cells, control mice were sacrificed, and BM cells magnetically sorted for CD19 with anti-APC magnetic beads (Miltenyi Biotec) according to the manufacturer’s instructions. Cells were plated in a round-bottom 96-well plate as 10^6^ cells/mL and incubated with increasing concentrations of SKi or vehicle (DMSO) and with IL-7 (1.25 ng/mL; Peprotech). Cell viability was measured using Annexin V/7AAD staining.

### Chemical screening

We screened Ba/F3 cells stably transduced with human *IL7R* mutant for effects on viability (measured by Alamar Blue) using the 80 compounds included in the InhibitorSelect™ 96-Well Protein Kinase Inhibitor Library II (Calbiochem). Analysis of the screening results was performed with cellHTS2 R package^83^, where each replicate compound measurement was normalized using the negative controls (DMSO-control) and converted to a *Z*-score.

### Mutagenesis of murine *Il7r* gene

The vector pUC19 harboring the wild-type murine *Il7r* cDNA (pUC19/Il7r WT) was used as template for mutagenesis, performed using the QuikChange II XL Site-Directed Mutagenesis Kit (Agilent Technologies) according to the manufacturer’s protocol. PCR amplifications were performed to introduce the p.S244_Val245insCPT using the mutagenic primer pair mIL7R-CPT-FWD and mIL7R_CPT_REV (Supplementary Data 17). The specific PCR conditions were the following: [denaturing phase 50 s at 95 °C; annealing phase 50 s at 60 °C; extension phase 5 min at 68 °C] × 18 cycles, thus generating the pUC19/*Il7r* CPT. Mutagenesis was confirmed by Sanger sequencing.

### Ba/F3 cell culture

Growth factor-dependent parental Ba/F3 cells or IL-7R-transduced Ba/F3 cells were maintained in culture medium (RPMI-1640 supplemented with 10% FBS, 2 mM l-glutamine, and penicillin/streptomycin). Parental Ba/F3 and *Il7r* WT transduced Ba/F3 cultures were supplemented with 2% (v/v) WEHI-3B-conditioned medium as source of murine IL-3 or 100 ng/mL of rhIL7 (Peprotech), respectively. Ba/F3 cells stably expressing wild type or mutant *Il7r* were deprived of IL-7 for 24 h prior the beginning of the experiments. Cells were then cultured in medium alone or supplemented with IL-7 (100 ng/mL) for the indicated time.

### Cell cycle analysis

Cells (1–2 × 10^6^) were resuspended in PBS, fixed and permeabilized with an equal volume of ice-cold 80% ethanol. Ribonuclease A (Sigma) was added at 50 mg/mL, and samples were incubated for 30 min at 37 °C. Propidium iodide (Sigma) was added at a final concentration of 2.5 mg/mL, and samples were analyzed by flow cytometry. Cell cycle distribution was determined using ModFit LT software (Verity).

### BrdU incorporation in vivo

Mice were intraperitonially injected with 10 mg/mL BrdU (BD Pharmingen) and after 12 h BM was collected and cells stained with fluorochrome-conjugated anti-CD45, IgM, CD19, IgD, and B220 antibodies. Then, cells were stained with APC-conjugated anti-BrdU antibody and the DNA and 7AAD, using the BrdU Flow Kit (BD Pharmingen), according to the manufacturer’s protocol.

### MTS assay

Cells were plated in a flat-bottom 96-well plate as 10^6^ cells/mL for the indicated time points. MTS (3-(4,5-dimethylthiazol-2-yl)-5-(3-carboxymethoxyphenyl)-2-(4-sulfophenyl)-2H-tetrazolium; Promega) was then added to each well (20 µL/well), and cells were kept at 37 °C for 4 h. Absorbance was measured at 490 nm using the Infinite M200 microplate reader (Tecan).

### Construction of mutant Kras expression vectors

The coding sequence of mutant murine Kras (Q61H) was PCR amplified from cDNA of spleen cells from an experimental mouse (from the model described above) harboring the *Kras* Q61H mutation using the forward and reverse primers described in Supplementary Data 17. The PCR product was subsequently digested with *Hpa*I and *Xho*I (New England Biolabs) and cloned into the *Hpa*I and *Xho*I sited of pMIG (Addgene 9044). The clones were verified by Sanger sequencing.

### Retroviral production

Retroviruses were produced using the pCLeco packaging vector and the pMig vector, either harboring the Q61H Kras mutation or mock, by co-transfection into HEK-293T cells, previously plated in a poly-d-lysine coating (Merck Life Sciences), using Lipofectamine 2000 (Life technologies) according to the manufacturer’s instructions. Subsequently, the viruses were concentrated using Amicon Ultra-15 Centrifugal Filter Unit with Ultracel-10 membrane from Merck Millipore and kept at −80 °C until usage.

### Retroviral transduction and transplantation of hematopoietic stem and progenitor cells

IL7R^mut^ and/or control mice were injected intraperitoneally with 300 μL of 5-fluorouracil (5-FU; 10 mg/mL), BM was collected 5 days after injection, and cells subjected to depletion of CD19 and CD3 with anti-APC magnetic beads (Miltenyi Biotec). Subsequently, cells were spin infected for 2 h at 1120 g at room temperature with 1 mL of viruses from either the pMig *Kras* Q61H or the pMig Empty vectors with 10 ng/mL of Polybrene (Merck Millipore). *Rag2*^−/−^*γ*_*c*_^−/−^ recipient mice were lethally irradiated (850 rad—two sub lethal irradiations) and reconstituted with 1.45 × 10^5^ transduced cells and 2 × 10^5^ stromal cells from Rag^−/−^γ_c_^−/−^ mice. BM of recipient mice was analyzed 5 weeks after injection.

### Statistical analysis

GraphPad Prism version 6-8.05 (GraphPad Software) was used to perform statistical analysis. Differences between groups were calculated using unpaired two-tailed *t*-tests. When appropriate, paired two-tailed *t*-tests and one-way ANOVA were used. Welsh’s correction was used in *t*-tests when variances were found different. Differences in the survival curves were calculated using log-rank Mantel–Cox test. *P* values lower than 0.05 were considered statistically significant. Tests used are indicated in figure legends.

### Reporting summary

Further information on experimental design is available in the Nature Research Reporting Summary linked to this paper.

## Supplementary information

Supplementary Information

Description of Additional Supplementary Files

Supplementary Data 1

Supplementary Data 2

Supplementary Data 3

Supplementary Data 4

Supplementary Data 5

Supplementary Data 6

Supplementary Data 7

Supplementary Data 8

Supplementary Data 9

Supplementary Data 10

Supplementary Data 11

Supplementary Data 12

Supplementary Data 13

Supplementary Data 14

Supplementary Data 15

Supplementary Data 16

Supplementary Data 17

Reporting summary

## Data Availability

The data supporting the findings from this study are available within the manuscript and the supplementary information. Source data are provided with this paper. In addition, WES and RNA-seq data are available from SRA under the accession number PRJNA718778, and proteomics data are available on PRIDE under the accession number PXD017147. Source data are provided with this paper.

## Source data

Source data
