# Supplementary material for: Interleukin-7 receptor α mutational activation can initiate precursor B-cell acute lymphoblastic leukemia
Source: Nat Commun. 2021 Dec 14;12:7268. doi: 10.1038/s41467-021-27197-5 (PMC8671594; doi:10.1038/s41467-021-27197-5)
Supplement: Supplementary file 1 — Supplementary Information [file 41467_2021_27197_MOESM1_ESM.pdf]

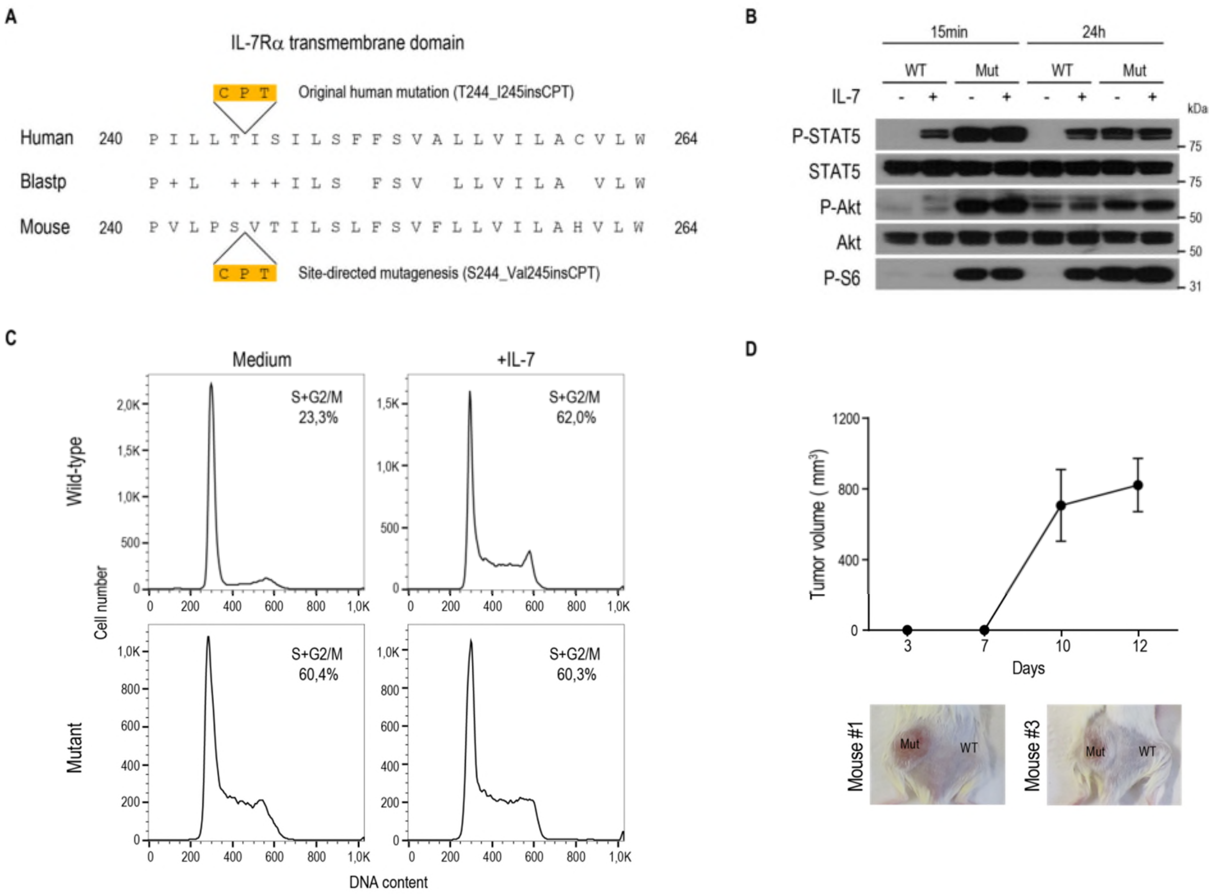

Supplementary figure 1. Generation of the constitutively active mutant murine *Il7r* and preliminary evaluation of its oncogenic potential. (A) Protein blast between human and murine IL-7Rα transmembrane domain. In the Blastp line exact matches are shown with the single-letter amino acid code, plus (+) sign for similar amino acids or otherwise left blank. In the human and murine lines the place of site-directed mutagenesis is noted. **(B)** Ba/F3 cells stably expressing wild-type or mutant Il-7rα were IL-7 deprived for 24h followed by short term (15min) and long term (24h) IL-7 stimulation (100ng/mL) and collected for immunoblot analysis of Jak/STAT5 (P-STAT5), PI3K/Akt (P-Akt) and mTOR (P-S6) signaling pathway activation. **(C)** Ba/F3 cells were treated as in (B) and collected at 48h for cell cycle analysis by flow cytometry. **(D)** NSG mice (n=8) were injected subcutaneously with Ba/F3 cells stably expressing murine *Il7r* WT (right flank) and the murine *Il7r* mutant (left flank). Tumors developed only the left flank. Graphic shows mean tumor volume ± SD. Images are representative of mice at the time of euthanasia. Source data are provided as a Source Data file.

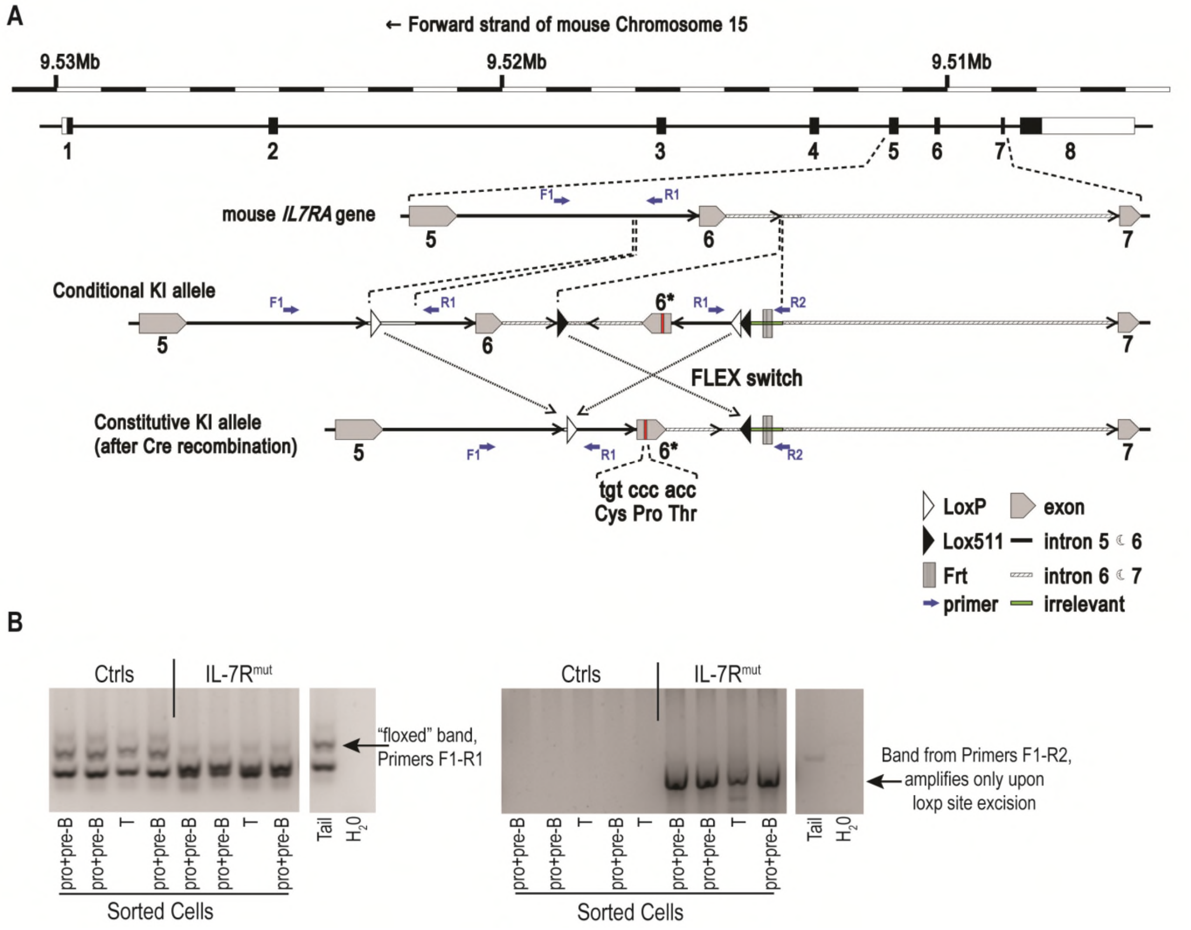

Supplementary figure 2. Generation of conditional knock-in of mutant *IL7R* in C57Bl6 mice. (A) FLEx switch strategy to generate the conditional mutant *IL7R* mice. **(B)** Genotyping (left) and verification of loxp site excision (right) PCR reactions with primers F1 / R1 and F1 / R2, respectively, were designed and performed on samples from sorted Pre+ProB or TCRβ^{pos} cells from control animals (CD2Cre^{neg}Il7R$^{cptfl/wt}$) or IL-7R^{mut} (CD2Cre^{pos}IL7R$^{cpt/wt}$) animals. Note the near absence of amplification of the floxed allele band (visible in the tail sample from one of the animals and in control samples) in IL-7R^{mut} samples in the genotyping PCR while the inversion/excision (FLEX switch) of the loxp sites allows primer pair F1 / R2 in verification reaction to amplify a band in loxp site excised DNA from the sorted cells. The band expected from the floxed allele was too big to be amplified in the PCR conditions used, as illustrated by the lack of amplification in the tail sample from one of the animals and in control Pro+PreB cells shown.

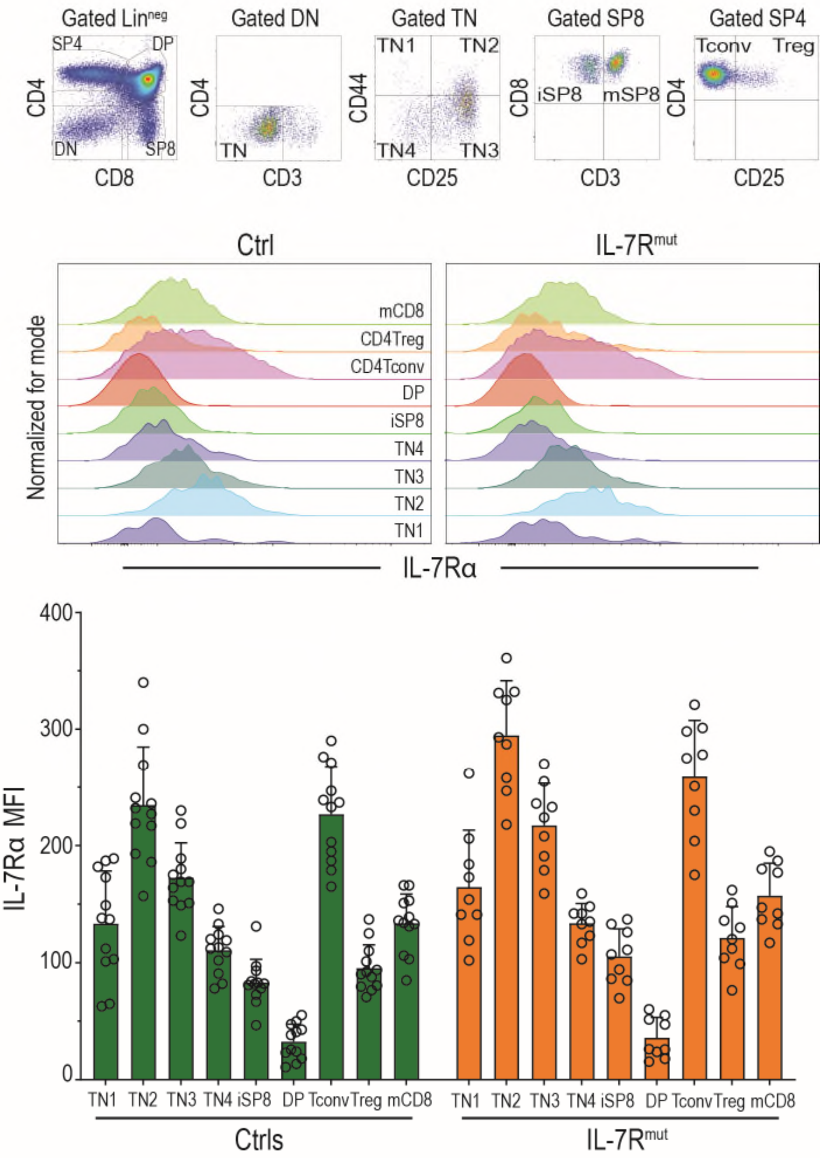

Supplementary figure 3. Thymic IL-7R expression is kept at normal levels and expression pattern maintained. Figure shows surface IL-7R expression in the thymus of 4 week old Control and IL7R^{mut} animals, as evaluated by flow cytometry. Dot plots show gating strategy used to define thymocyte subpopulations and histograms depict IL-7R expression in each of the populations in control and IL-7R^{mut} animals. Plots below shows analysis in all animals analyzed (Control: n=12; IL-7R^{mut}: n=9). Mean ± standard deviation are also shown. Expression pattern shows normal regulation of IL-7R expression throughout thymic development. The antibody recognizes both the wild type and mutant IL-7Rα, as depicted in Supplementary figure 5. Source data are provided as a Source Data file.

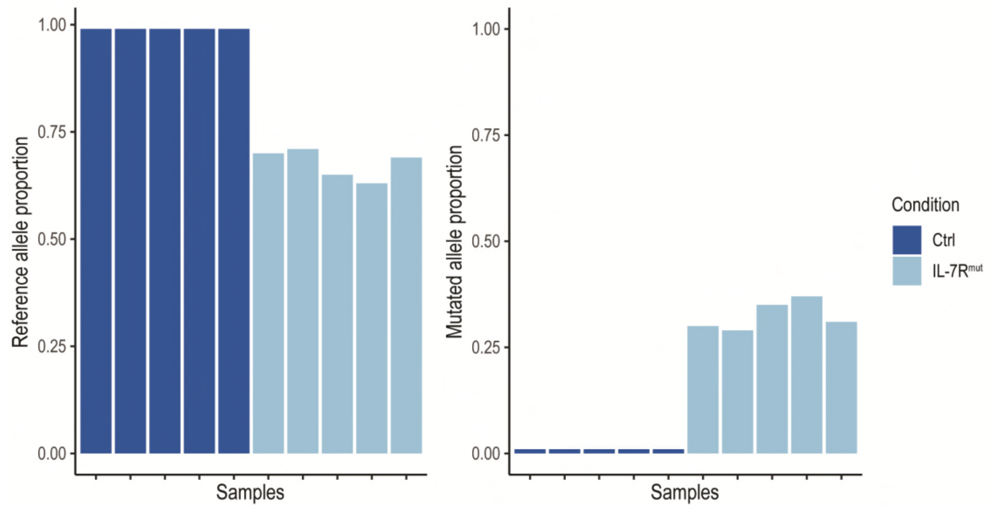

Supplementary figure 4. Relative expression of the reference (wild-type) and mutant *IL7R* alleles in control and pre-leukemic mice. Source data are provided as a Source Data file.

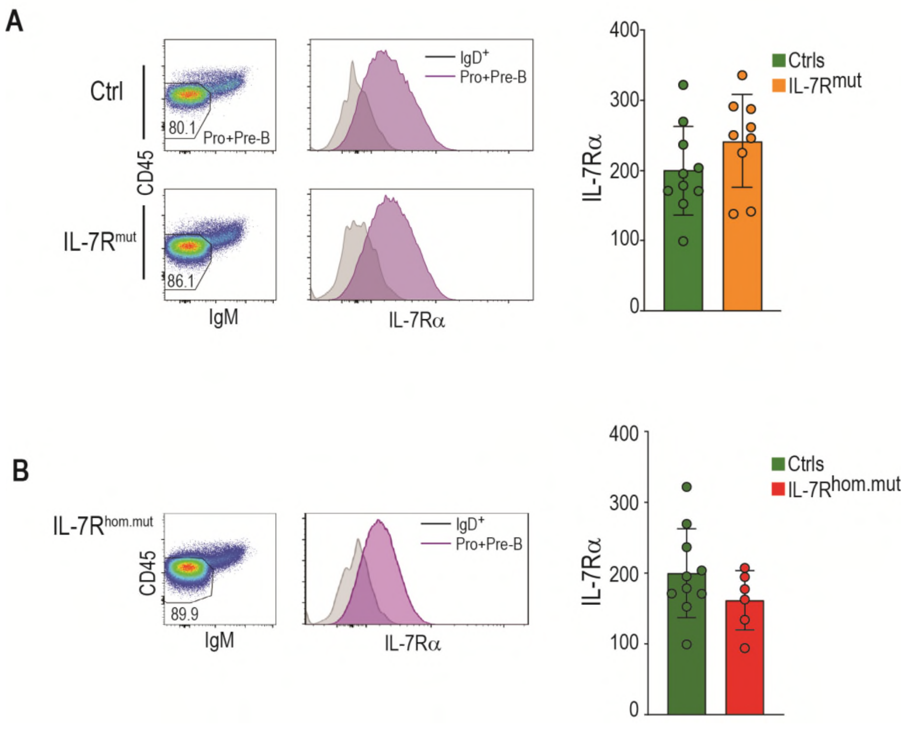

Supplementary figure 5. Bone marrow B-cell precursor IL-7R expression is kept at normal levels and expression pattern maintained. (A) Surface IL-7R expression in the bone marrow B cell lineage cells (gated $CD19^+$) of 4 week old Control and $IL7R^{mut}$ animals, as evaluated by flow cytometry. Dot plots show gating strategy used to define pro+pre-B cells. Bar plots summarize data from all animals analyzed with mean ± standard deviation (Control: n=10; $IL\text{-}7R^{mut}$: n=9). IL-7Rα expression is shown as MFI difference to IgD^+ cells in the same mouse (IgD^+ cells were used as negative controls since they do not express IL-7Rα). Expression pattern shows normal regulation of IL-7R in bone marrow B lineage cells. **(B)** The antibody recognizes also mutant IL-7Rα. To ensure that the IL-7Rα signal in heterozygous *IL7R* mutant samples is not due merely to the detection of the wild type receptor, we also evaluated IL-7Rα levels in homozygous *IL7R* mutant mice (which do not express the wild type allele). Bar plots summarize data from all animals analyzed ± SD (Control: n=10; $IL\text{-}7R^{hom.mut}$: n=6). No statistically significant differences were detected. Student's two-tailed unpaired t-test. Source data are provided as a Source Data file.

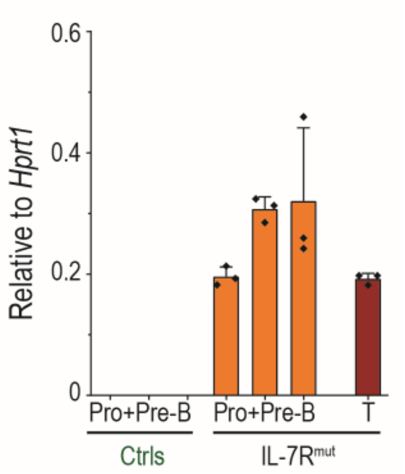

Supplementary figure 6. Mutant *Il7r* expression is similar in B and T cell lineages. Figure shows qRT-PCR analysis performed in triplicate on BM sorted pro+pre-B cells from Control (n=3) and IL7R^{mut} animals (n=3) and spleen T cell sorted cells from IL7R^{mut} animals (n=1), with custom Taqman probes designed for detection of mutant *Il7r* only. Mean ± standard deviation are also shown. Note the absence of signal in Control samples, and similar expression levels in B cell precursor and T cell samples from IL-7R^{mut} animals. Source data are provided as a Source Data file.

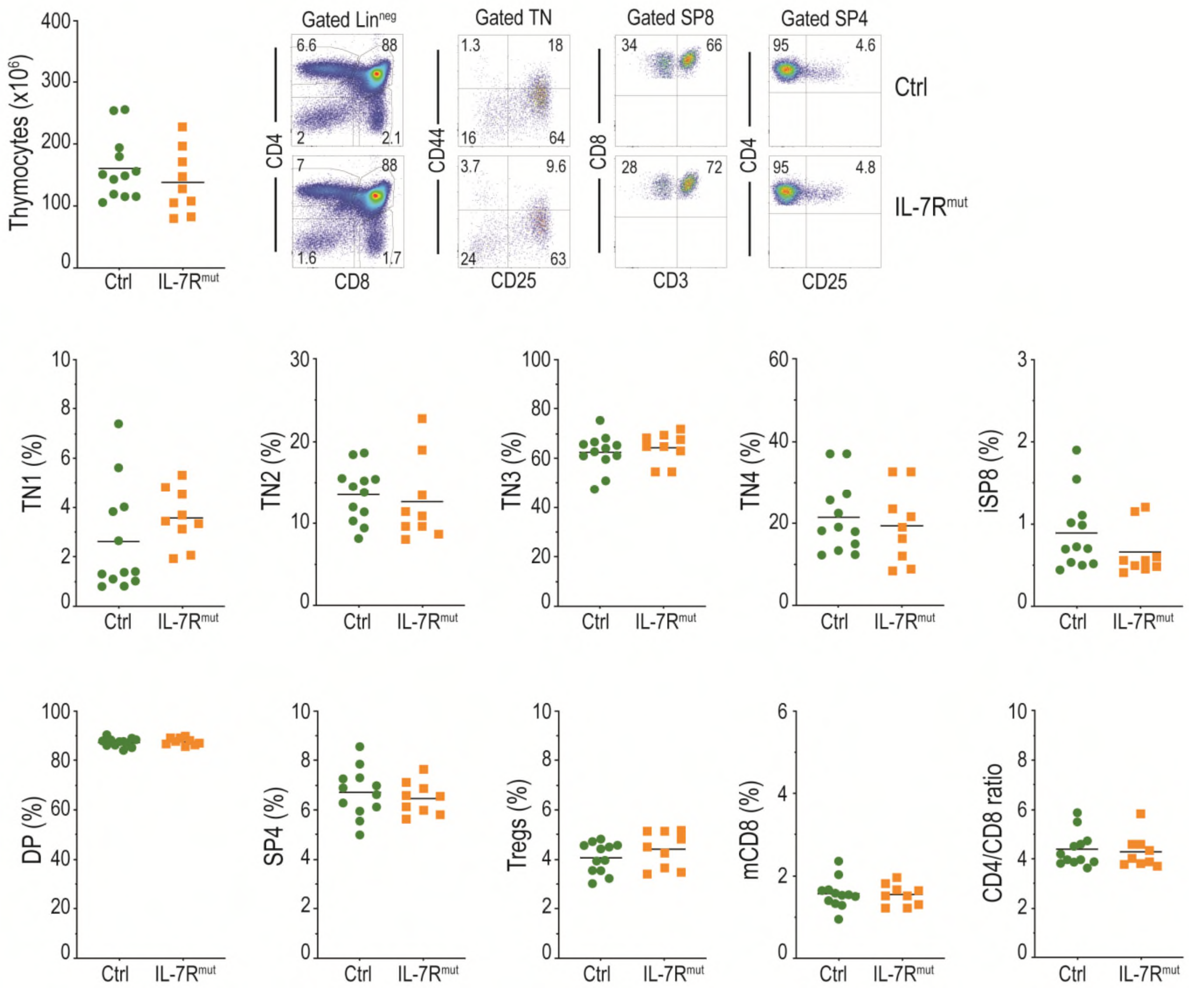

Supplementary figure 7. Normal thymic T cell development in IL7Rmut animals. The figure shows analysis of 4 week-old animal thymus from Control (n=12) and IL-7R^{mut} groups (n=9). Total thymocyte numbers are shown and dot plots depict a representative thymus from each group and gating strategy used. Numbers inside plots are frequencies within respective gate or regions. Scatter plots are results from all animals and show virtually no impact in thymocytes from IL-7R^{mut} animals. Source data are provided as a Source Data file.

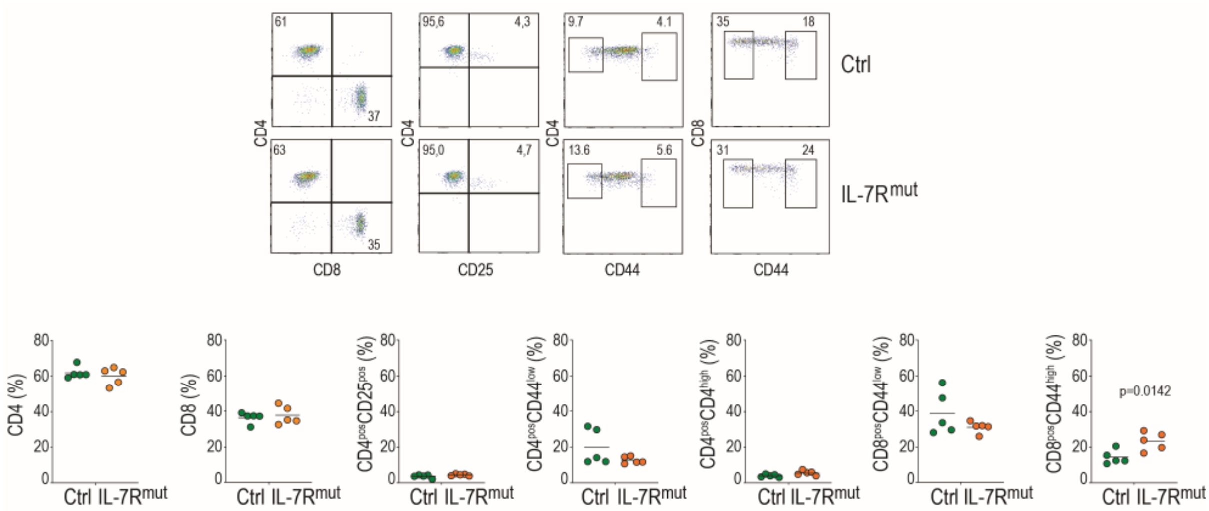

Supplementary figure 8. Peripheral T cells in young IL7R^{mut} animals are normal. 6 week old Control (n=5) or IL-7R^{mut} animals (n=5) were bled and T cell subpopulations evaluated by flow cytometry, with CD4^{pos} T cells subdivided in regulatory (Tregs), naïve (Cd44^{low}) and memory (CD44^{high}) and CD8^{pos} T cells subdivided in naïve (Cd44^{low}) and memory (CD44^{high}). No differences were observed with the only exception a mild increase in memory CD8 T cells in the IL-7R^{mut} animals. Numbers inside plots are frequencies within respective gate or regions. Student's two-tailed unpaired t-test. Source data are provided as a Source Data file.

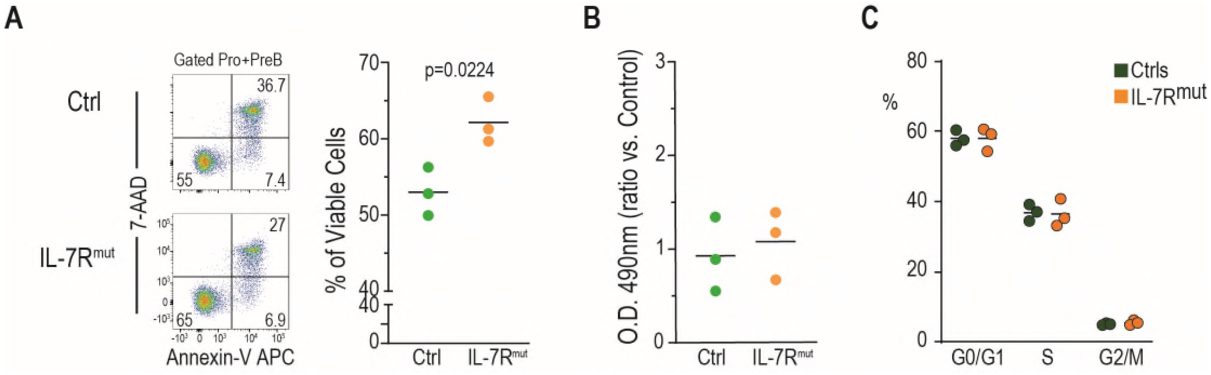

Supplementary figure 9. Pre-leukemia cells display higher viability, but not proliferation, than normal pro- and pre-B cells. Sorted pro+pre-B cells from control (n=3) and IL7R^{mut} donors (n=3) were cultured in triplicate wells (10^5/well) for 24 hours in RPMI medium without IL-7. **(A)** Cells were collected and viability determined by Annexin-V /7-AAD staining. Shown is a representative well from one animal from each group, gated on pro+pre-B cells (IgM^{neg}CD45^{int}). Data from the 3 animals is shown in the graph on the right (mean value from triplicate wells). Two-tailed unpaired student's t-test. **(B)** Cells were incubated with MTS for 4 hours and absorbance was determined at 490nm. Absorvance values were subtracted to medium control for background correction. Average from each individual donor was then compared to mean value from control group and value is depicted (3 control animals; 3 IL7R^{mut} donor animals). **(C)** 5-week old animals from control and IL-7Rmut (n=3 for each group) were injected i.p. with BrdU (10mg/mL). 12 hours later, animals were sacrificed and bone marrow collected for analysis. Cell cycle analysis of pro+pre-B cell population in bone marrow cells from animals stained with anti-BrdU antibody and 7-AAD was performed. Cell cycle distribution is shown. Source data are provided as a Source Data file.

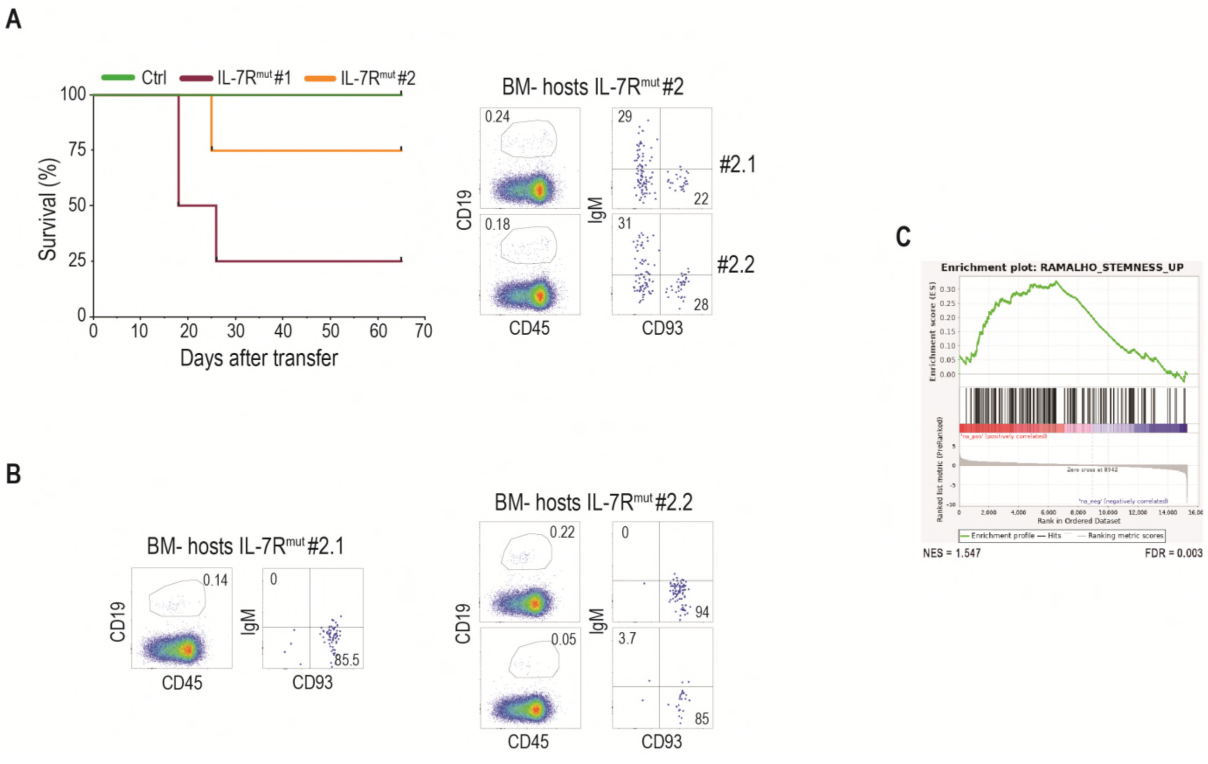

Supplementary figure 10. Self-renewal in IL7R^{mut} B cell precursors. (A-B) In order to determine whether mutational driven IL-7R activation leads to self-renewal or developmental block in B cell precursors, 2x10^{6} pro+pre-B cells were FACS sorted from the BM of 8 week old control or IL-7R^{mut} animals and transferred into sub-lethally irradiated (400rads) B6Rag2$^{-/-}\gamma_c^{-/-}$ hosts. Animals were monitored for up to 66 days, when surviving animals were sacrificed and BM was analyzed for the presence of B cell precursors as evaluated by CD19 and CD93 expression. From these animals, 10x10^{6} total BM cells were collected and transferred into a group (n=4) of sub-lethally irradiated secondary hosts, followed for 90 days and then analyzed for the presence of B-lineage cells in the BM and spleen. **(A)** Survival curves show leukemia-driven lethality in hosts of IL-7R^{mut} pro+pre-B cells but not in animals receiving Control pro+pre-B cells, whereas dot plots show BM analysis of surviving hosts of IL-7Rmut#2 BM cells, showing the presence of a relevant proportion of CD93^{+}IgM^{-} cells. Numbers inside plots are frequencies within respective gate or regions. **(B)** Dot plots show analysis of secondary hosts of BM precursors at day 90 after transfer, showing a clear population of CD93^{+}IgM^{-} precursor cells in these animals. Numbers inside plots are frequencies within respective gate or regions. Note the absence of IgM^{+} B-linage cells, demonstrating not only the self-renewing capacity of the IL-7R^{mut} precursors but also a developmental block prior to expression of IgM. No B cells were found in the spleen and disease did not ensue in any of the hosts. **(C)** Gene set enrichment analysis (GSEA)-enrichment plot of differential gene expression between pre-leukemia and control samples, prior to transplant, for genes involved in stemness signatures (Ramalho et al, Science 2002). Source data are provided as a Source Data file.

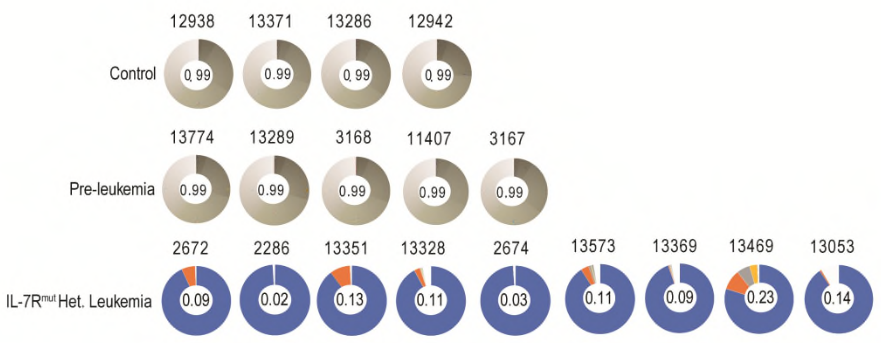

Supplementary figure 11. Clonality pie charts, based on *IgH* sequencing, of all animals analyzed - of which a selection is shown in Fig. 2c. Each colored slice corresponds to a clone, indicative of clonality. Grey areas correspond to many rare clones, indicative of polyclonality. Equitability values (ranging from 0 to 100, inversely proportional to clonality) are shown in the center of the pie charts. See methods for further details. Source data are provided as a Source Data file.

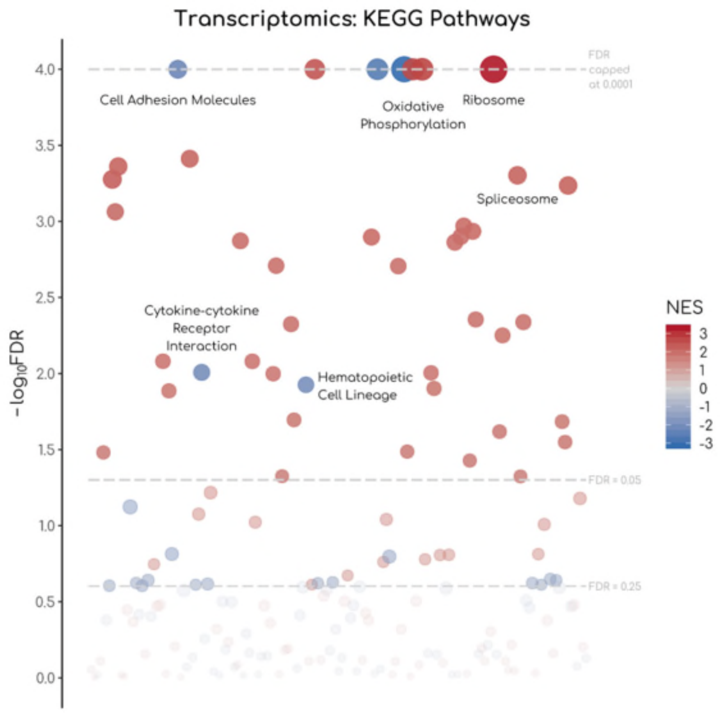

Supplementary figure 12. Gene set enrichment analysis (GSEA) of KEGG pathways considering differential gene expression between IL-7R^{mut} mice and controls. Source data are provided as a Source Data file.

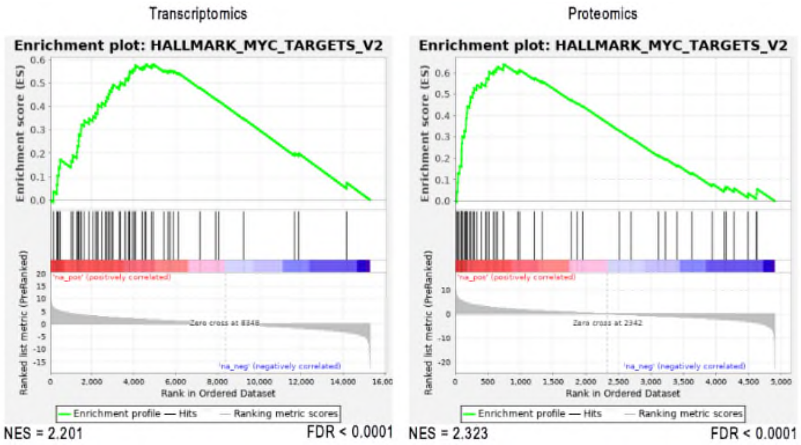

Supplementary figure 13. Gene set enrichment analysis (GSEA) of transcriptomic and proteomic differences between IL-7R^{mut} mice and controls shows a significant (FDR<0.05) enrichment (NES>1) of the MYC targets (V2) hallmark gene set.

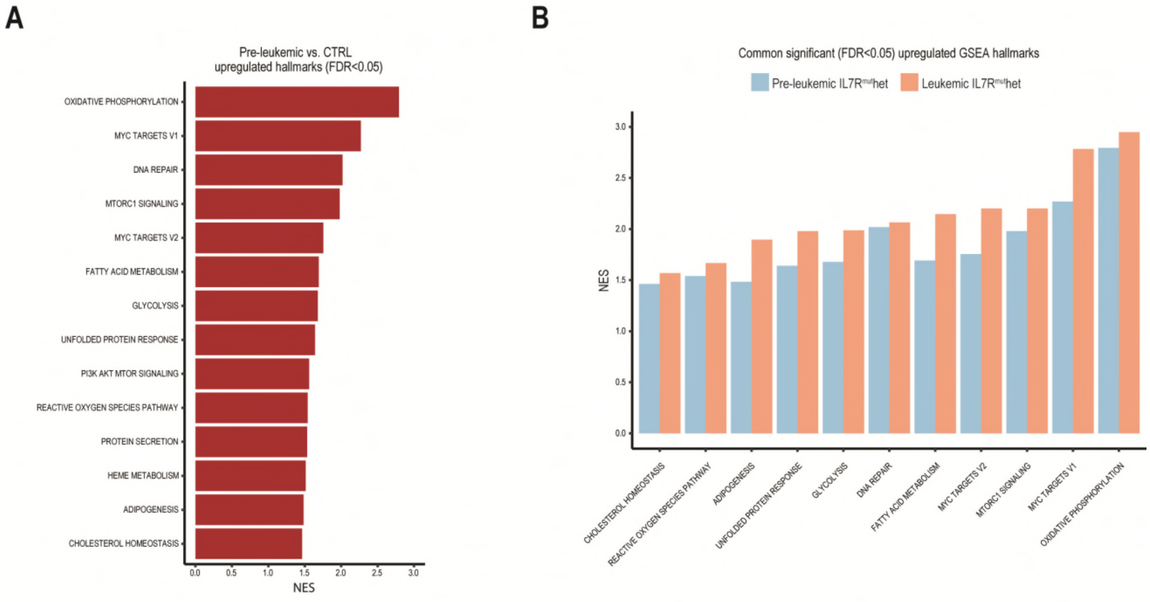

Supplementary Figure 14. Further transcriptomics characterization of pre-leukemias and leukemias as compared to healthy B-cell precursors. Related to Figure 4. **(A)** Significantly upregulated gene set enrichment analysis hallmarks in pre-leukemic IL7R^{mut}het samples. **(B)** Normalized enrichment score (NES) of common significantly upregulated hallmarks in pre-leukemic and leukemic IL7R^{mut}het samples. Source data are provided as a Source Data file.

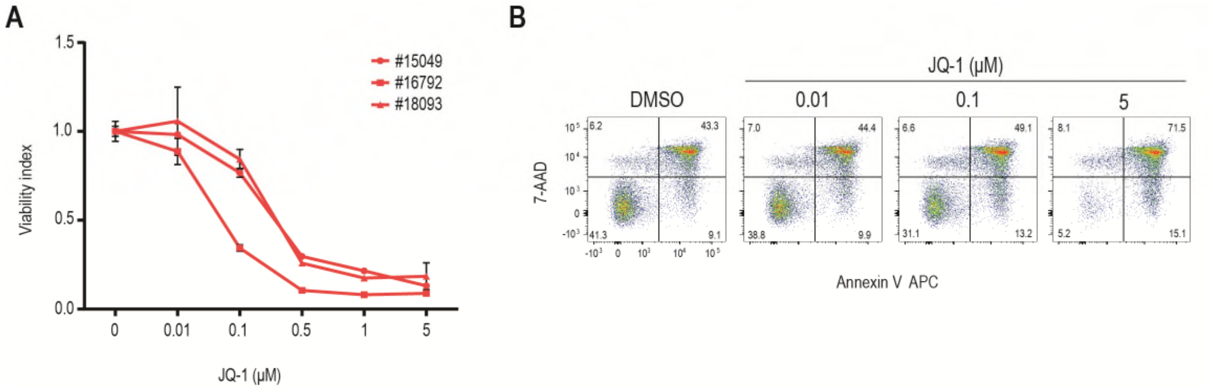

Supplementary Figure 15. Dose-dependent effect of the BET bromodomain inhibitor JQ1 on the viability of IL7Rmut leukemias *in vitro*. (A) Viability index of 3 independent leukemia samples incubated for 24 hours in medium with vehicle control (DMSO) or increasing concentrations of JQ1. Each condition was performed in triplicates. Mean±standard deviation are depicted. Viability index = frequency of viable cells in each experimental condition/frequency of viable cells in the control condition. **(B)** Representative plots from one of the leukemias. Numbers in plots denote frequency in the respective quadrant. Source data are provided as a Source Data file.

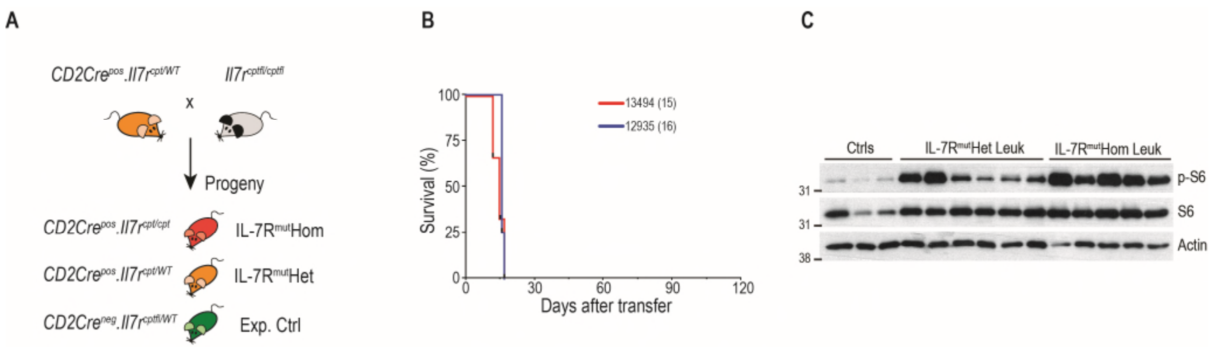

Supplementary figure 16. (A) Scheme illustrating crossing performed in order to obtain IL-7Rmut Homozygous animals. **(B)** Leukemic cells ($2x10^5$) from IL-7R^{mut}Hom animals were transferred into B6Rag$_2^{-/-}\gamma_c^{-/-}$ hosts and animals were closely monitored. As disease developed, survival curves depict the capacity to transfer disease by the transferred leukemic cells. **(C)** Shown are WB results for the analysis of pro+pre-B cells sorted from control (n=3) and leukemic cells sorted from IL-7R^{mut}Het (n=6) or IL-7R^{mut}Hom (n=5), followed by immunoblot analysis of mTOR pathway activation. Note the stronger signal in p-S6 in homozygous leukemias even in comparison with the majority of the heterozygous leukemias. Same western blots as shown in figure 4f. Source data are provided as a Source Data file.

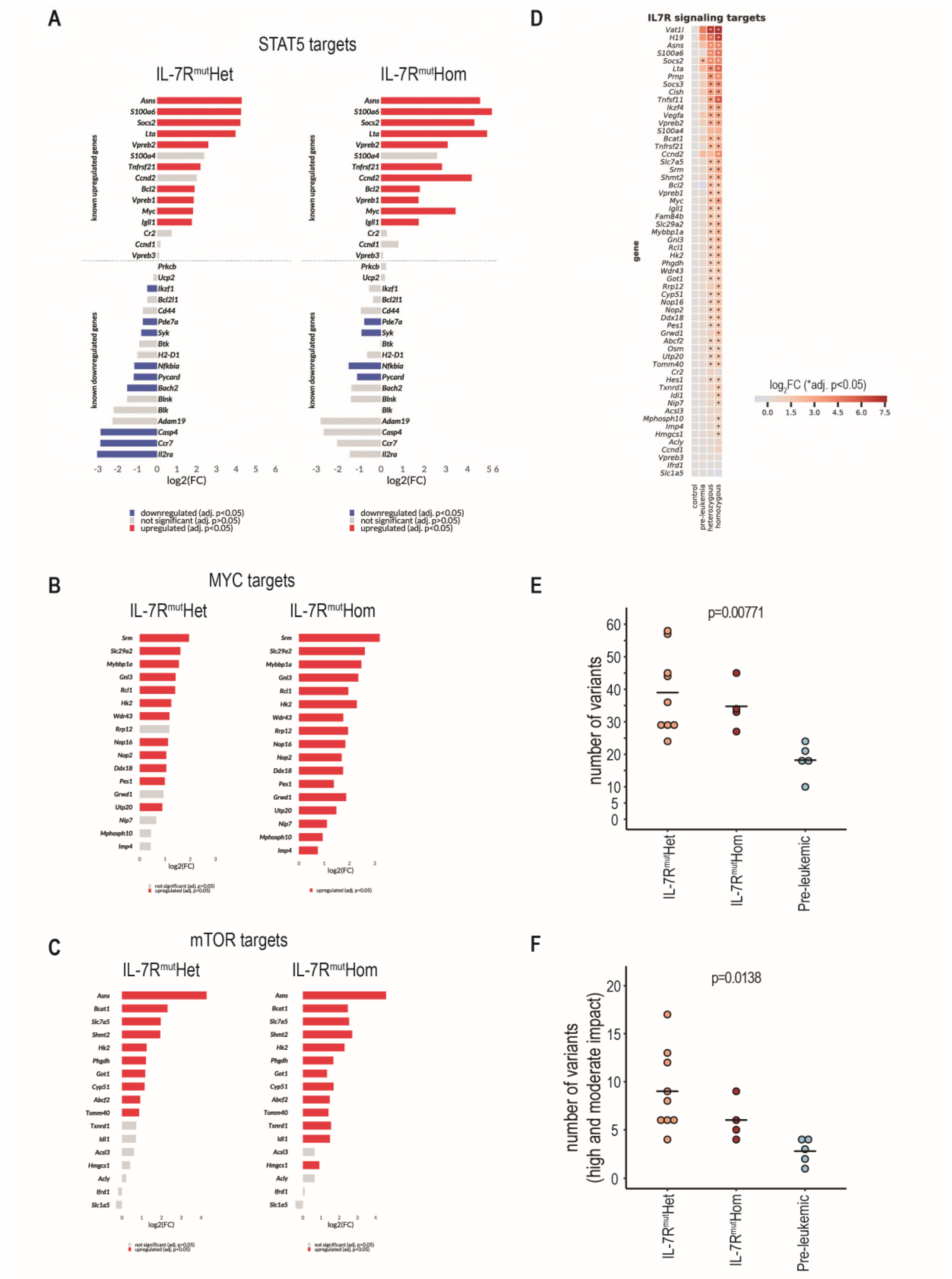

Supplementary figure 17. Differential expression of **(A)** STAT5, **(B)** MYC, and **(C)** mTOR targets in IL-7R^{mut}Het and IL-7R^{mut}Hom mice when compared to control samples, significantly upregulated (adj. $p<0.05$; moderate t-test) genes are shown in red, significantly downregulated (adj. $p<0.05$) in blue. **(D)** Heatmap of differential gene expression of known IL-7R signaling targets in pre-leukemic and IL-7R^{mut}Het and IL-7R^{mut}Hom mice compared to controls (reference category). **(E)** Total number of genetic variants (single nucleotide variants (SNVs) and indels) in IL-7R^{mut}Het leukemias (n=9), IL-7R^{mut}Hom leukemias (n=4) and pre-leukemias (n=5). One-way ANOVA. **(F)** Variants with predicted high or moderate impact in IL-7R^{mut}Het leukemias (n=9), IL-7R^{mut}Hom leukemias (n=4) and pre-leukemias (n=5). One-way ANOVA. Related to figures 4 and 5. Source data are provided as a Source Data file.

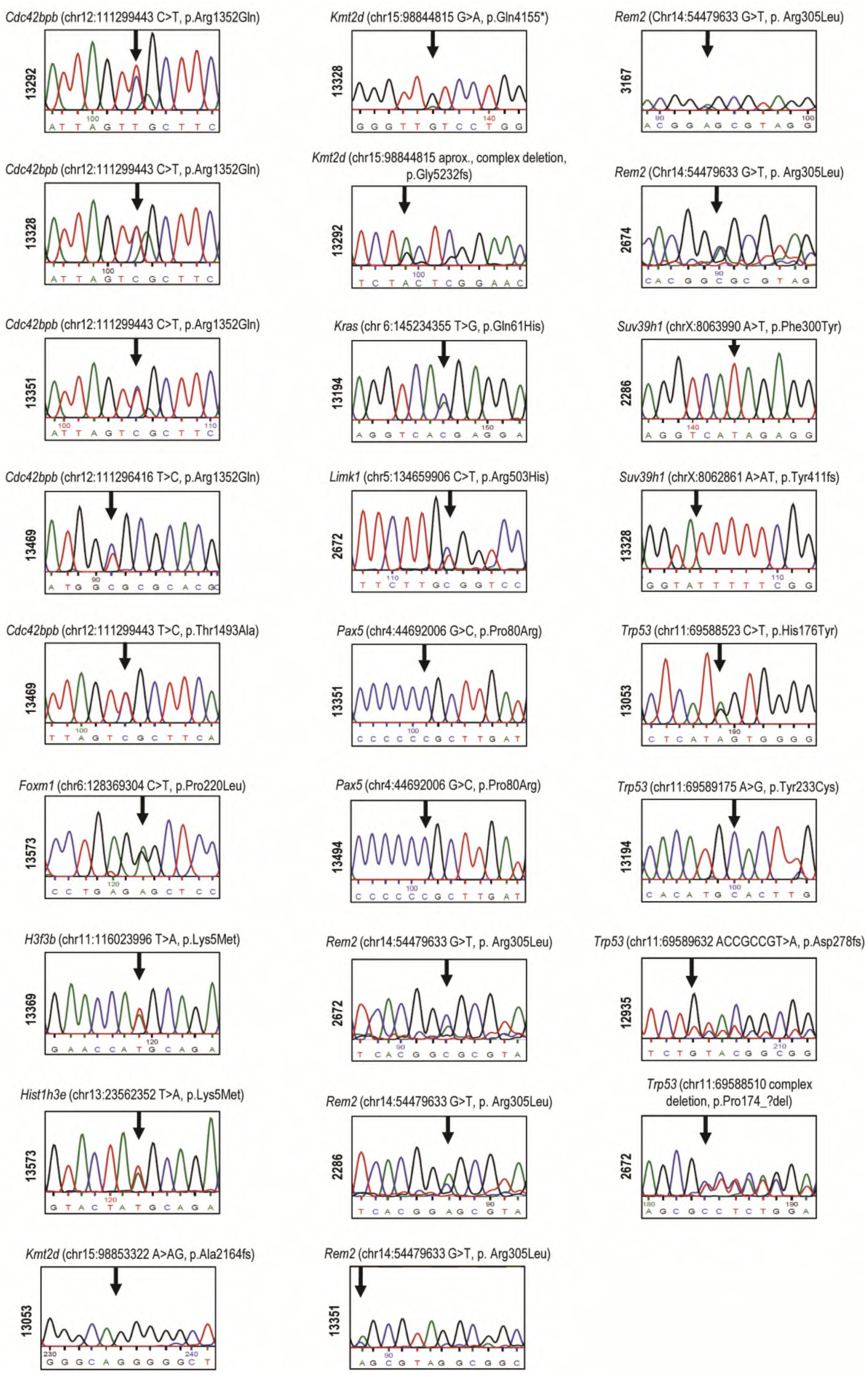

Supplementary figure 18. Sanger sequencing validation of variants in the indicated genes identified through whole-exome sequencing (WES). Related to Figure 6b.

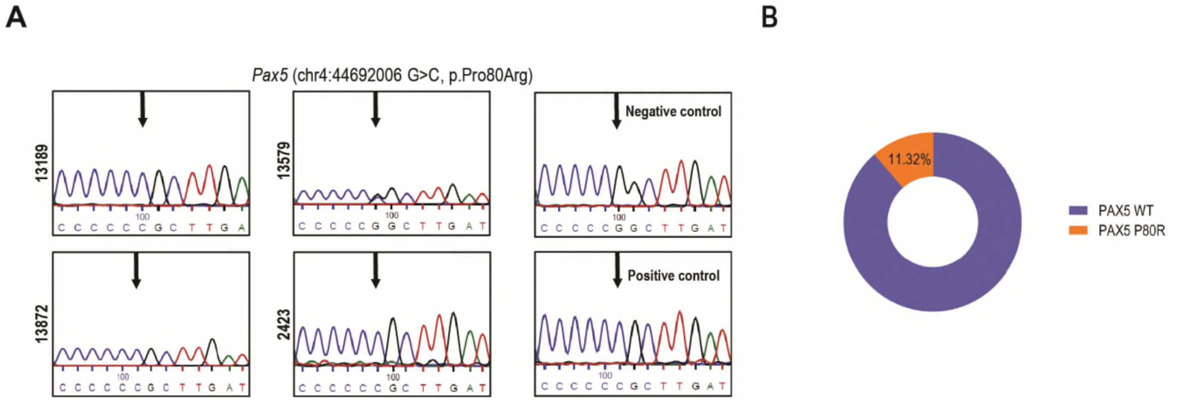

Supplementary figure 19. Overall frequency of PAX5 P80R cases. (A) Additional PAX P80R samples identified by Sanger sequencing. (B) Frequency of PAX5 P80R cases identified out of n=53 cases analyzed.

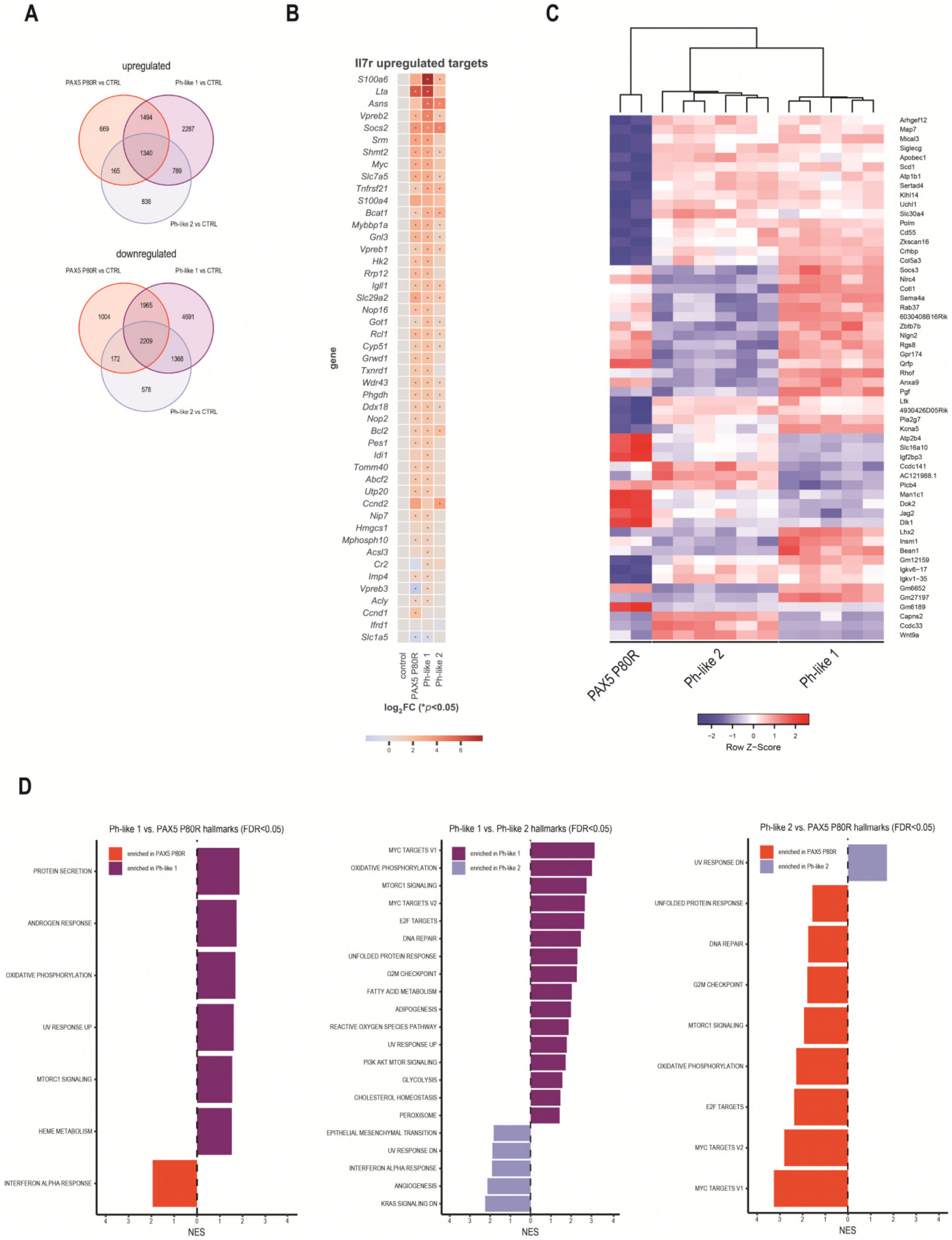

Supplementary figure 20. Further transcriptomics characterization of Ph-like 1, Ph-like 2, and PAX5 P80R subgroups. (A) Venn diagrams of common significantly upregulated or downregulated genes of each subgroup when compared with controls. **(B)** Heatmap of differential gene expression of known IL-7R signaling targets in PAX5 P80R, Phlike-1 and Phlike-2 subgroups compared to controls (reference category; moderate t-test). **(C)** Heatmap of the top 25 most significant differentially expressed genes between the three comparisons (Ph-like 1 vs. Ph-like 2, Ph-like 1 vs. PAX5 P80R, Ph-like 2 vs. PAX5 P80R, n = 56 total, as common genes are represented only once). **(D)** Significant (FDR<0.05) hallmarks between groups assessed through gene set enrichment analysis. Source data are provided as a Source Data file.

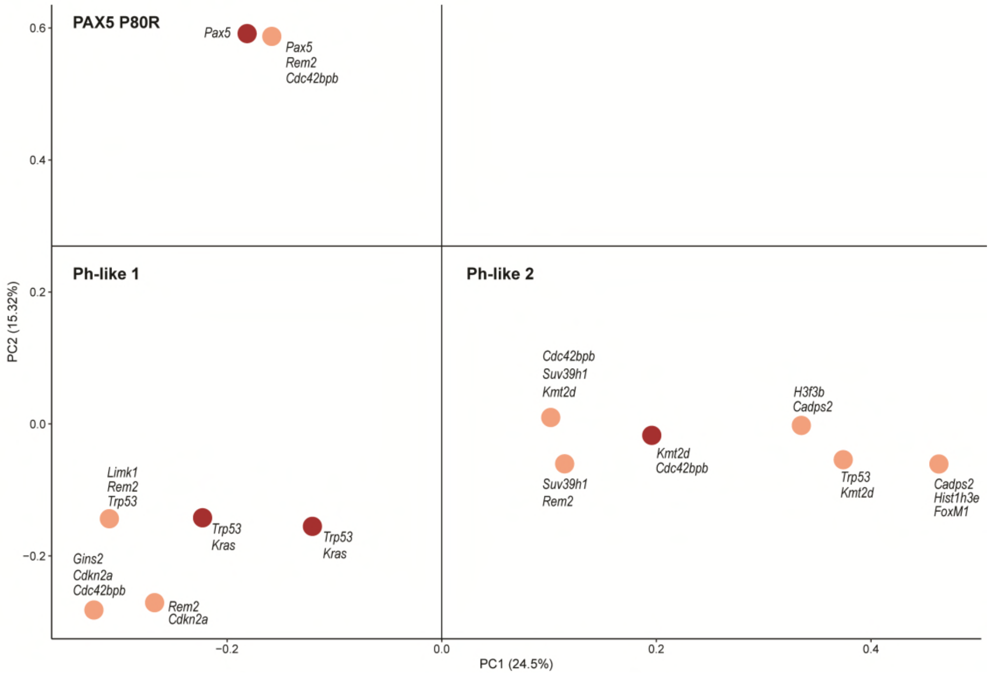

Supplementary figure 21. Overlay of the genes of interest (figure 6b) with the principal component analysis (PCA) plot of gene expression profiles from IL-7R^{mut}Het (in salmon) and IL-7R^{mut}Hom (in red) mice (figure 6a) and the apparent separation of transcriptomic groups based on genetic alterations. Source data are provided as a Source Data file.

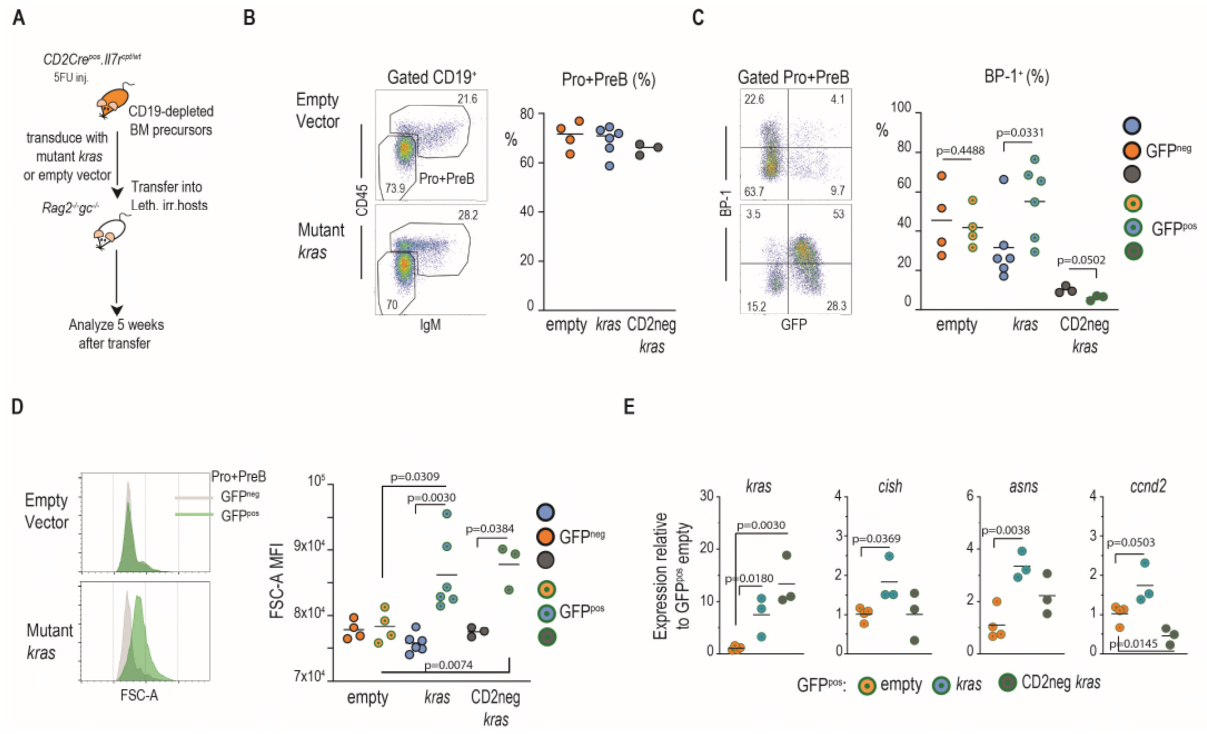

Supplementary figure 22. *Kras* mutation cooperates with mutant *IL7R*. **(A)** Scheme of experimental design to evaluate the impact of Kras mutations in mutant IL-7R driven leukemia development. CD19- and CD3-depleted BM precursors from 5FU-injected CD2Cre^{pos}.Il7r$^{cpt/wt}$ animals (hence, *IL7R* mutant) were spin infected with pMIG Kras Q61H or pMIG empty vectors and 1.4x10^{5} cells were then transferred into lethally irradiated *Rag2*$^{-/-}$ *Il2rg*$^{-/-}$ (*Rag2*$^{-/-}$*gc*$^{-/-}$) hosts (Kras, n=7; pMIG empty, n=7 animals). Hosts were euthanized 5 weeks after transfer. In parallel, the same procedure was performed with BM precursors from 5FU-injected CD2Cre^{neg}.Il7r$^{cpt/wt}$ (hence, *IL7R* wild type), spin infected with pMIG Kras Q61H vector (n=3). **(B)** Representative dot plots of CD19pos cells from the BM of animals in the experiment described in (A), 5 weeks after transfer, showing B cell precursor gating (left) and values for Pro+PreB cell fraction for each individual mouse (right): CD2Cre^{pos}.Il7r$^{cpt/wt}$ pMIG empty (empty), CD2Cre^{pos}.Il7r$^{cpt/wt}$ pMIG Kras Q61H (*kras*) and CD2Cre^{neg}.Il7r$^{cpt/wt}$ Kras Q61H (CD2^{neg} *kras*). **(C)** Expression of BP-1/ENPEP against GFP in a representative animal from the same groups (left), with data for all animals summarized in graph (right). **(B,C)** Numbers in dot plots in are frequencies within the indicated gates or quadrants. Paired two-tailed student's t-tests (GFP^{pos} vs GFP^{neg} in the same individual) were performed and p-values are shown. Only 6 animals were analyzed in the *kras* (CD2Cre^{pos}.Il7r$^{cpt/wt}$ CD2Cre^{pos} pMIG Kras Q61H) group because one of the animals died of leukemia before the end of the experiment, as depicted in Supplementary figure 23. **(D)** Histograms (left) depict FSC-A in GFP^{pos} (green) and GFP^{neg} (gray) Pro+Pre B cells from a representative animal from the same groups as in (B,C). Scatter plot graph (right) shows FSC MFI in all animals from the experiment. Paired t-tests (GFP^{pos} vs GFP^{neg}) were used to compare within each group while unpaired t-tests were used when comparing GFP^{pos} groups from CD2^{neg} (*IL7R* wild type) and CD2^{pos} (*IL7R* mutant) Kras-transduced precursor groups to empty vector (CD2^{pos}; *IL7R* mutant) group. P-values are shown. **(E)** Gene expression analysis of *Kras*, *Cish*, *Asns* and *Ccnd2* was performed by qRT-PCR in sorted GFP^{pos} Pro+Pre-B-cells collected from the bone marrow from animals from each group: empty (n=4), *kras* (n=3) and CD2^{neg} *kras* (n=3). Unpaired two-tailed t-tests. Source data are provided as a Source Data file.

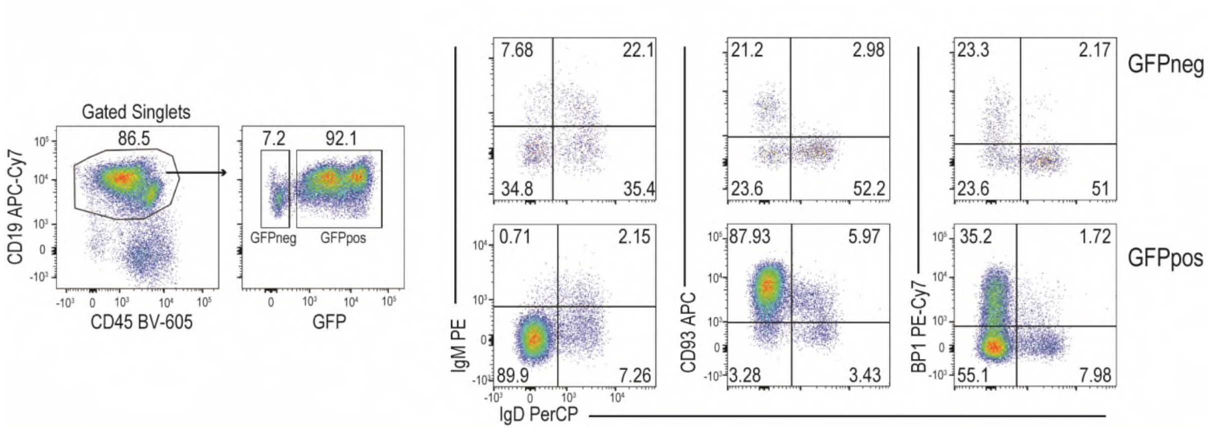

Supplementary figure 23. One of the animals transplanted with IL7R mutant Kras Q61H-expressing B-cell precursors developed ALL. From the experiment described in Supplementary figure 22, one of the animals receiving pMIG Kras Q61H transduced BM precursors developed overt signs of disease 31 days after transfer and was humanely euthanized. Depicted here is the flow cytometry analysis of BM cells, showing the presence of GFP^{pos} leukemic cells, expressing precursor markers CD93 and BP-1, in contrast with GFP^{neg} cells in the same animal displaying a higher fraction of IgM and IgD $CD19^{+}$ lymphocytes.

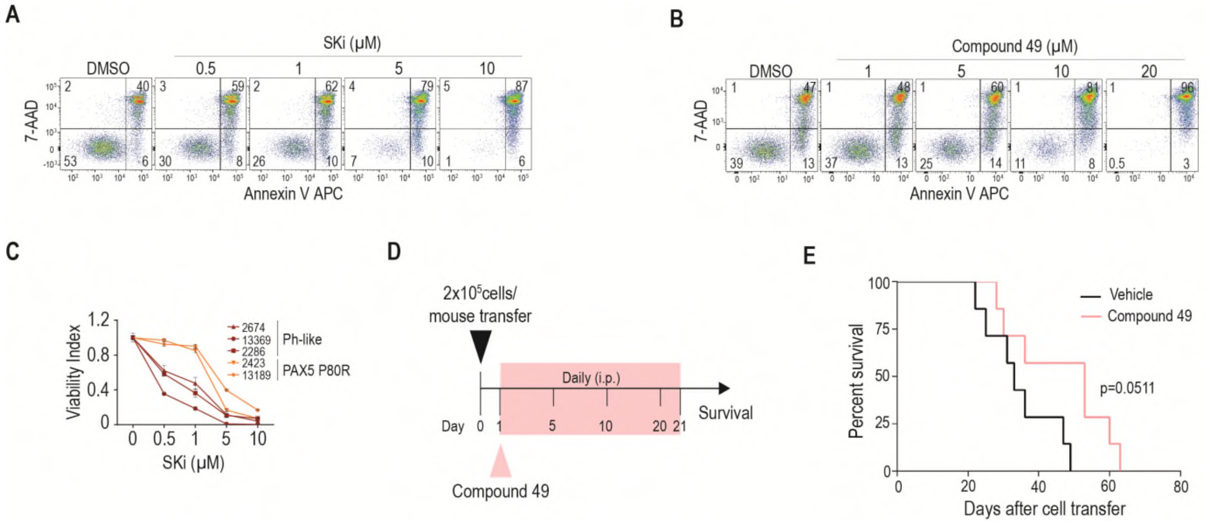

Supplementary figure 24. *In vitro* and *in vivo* effects of SK inhibitors on mouse IL-7Rmut leukemias. Complementary to Figure 7. **(A,B)** Viability (Annexin V/7AAD expression) of representative leukemia cells incubated for 12 hours with DMSO vehicle control or increasing concentrations of **(A)** SKi or **(B)** compound 49. **(C)** Dose-dependent effect of SKi on cell viability of PAX5 P80R and Ph-like leukemias. Each condition was performed in triplicates for each sample. Mean±sem are depicted. Viability index = frequency of viable cells in each experimental condition/frequency of viable cells in the control condition. **(D)** Compound 49 *in vivo* administration scheme. Leukemia cells ($2x10^5$) were transferred into Rag-/-γc-/- hosts. **(E)** Kaplan-Meyer survival curves, and respective LogRank p value, of animals (n=7 each group) treated with vehicle or PLR-11 (5 mg/Kg). Source data are provided as a Source Data file.

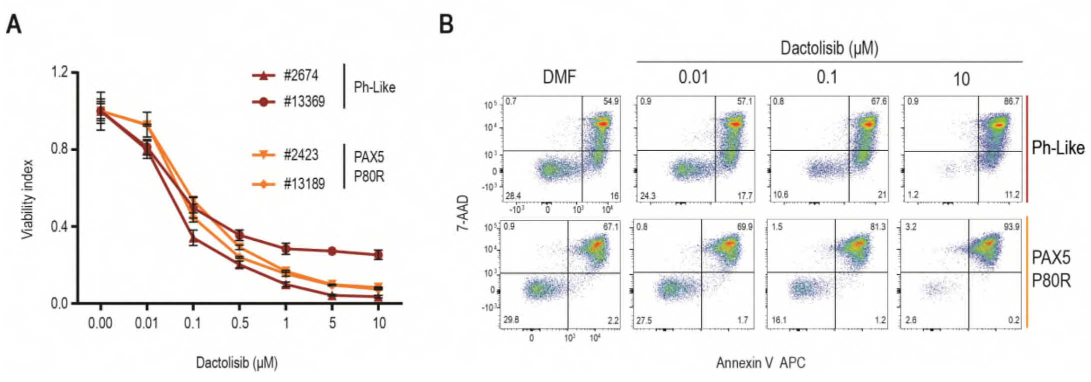

Supplementary Figure 25. *In vitro* effects of Dactolisib on mouse Ph-like and PAX5 P08R IL-7Rmut leukemias. (A) Viability index of 4 independent leukemia samples (2 PAX5 P80R and 2 Ph-like) incubated for 12 hours in medium with vehicle control (DMSO) or increasing concentrations of Dactolisib. Each condition was performed in triplicates. Mean±sem are depicted. Viability index = frequency of viable cells in each experimental condition/frequency of viable cells in the control condition. **(B)** Representative plots from one leukemia of each subtype. Numbers in plots denote frequency in the respective quadrant. Source data are provided as a Source Data file.

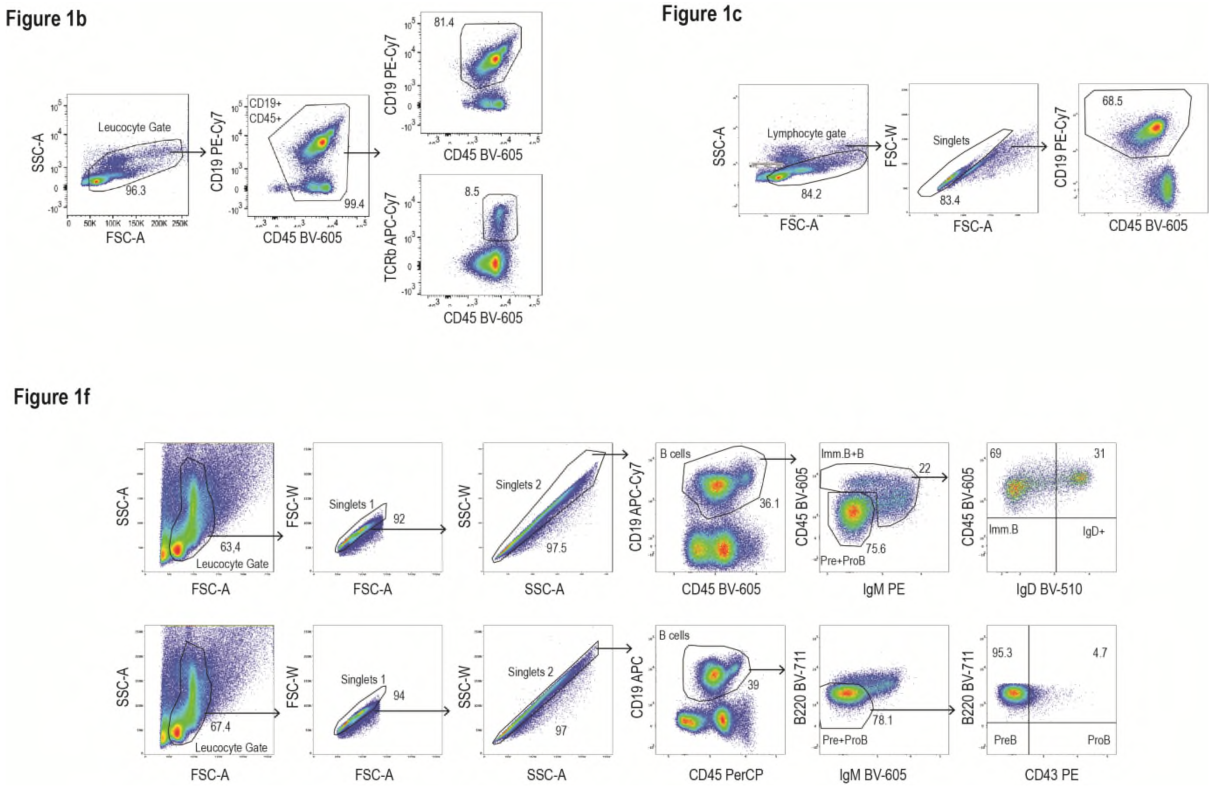

Supplementary Figure 26. Gating strategy for flow cytometry data in figure 1. Representative examples of the gating strategies used in the flow cytometry data presented in the indicated figure panels. Numbers in plots correspond to the frequency in the respective gate.

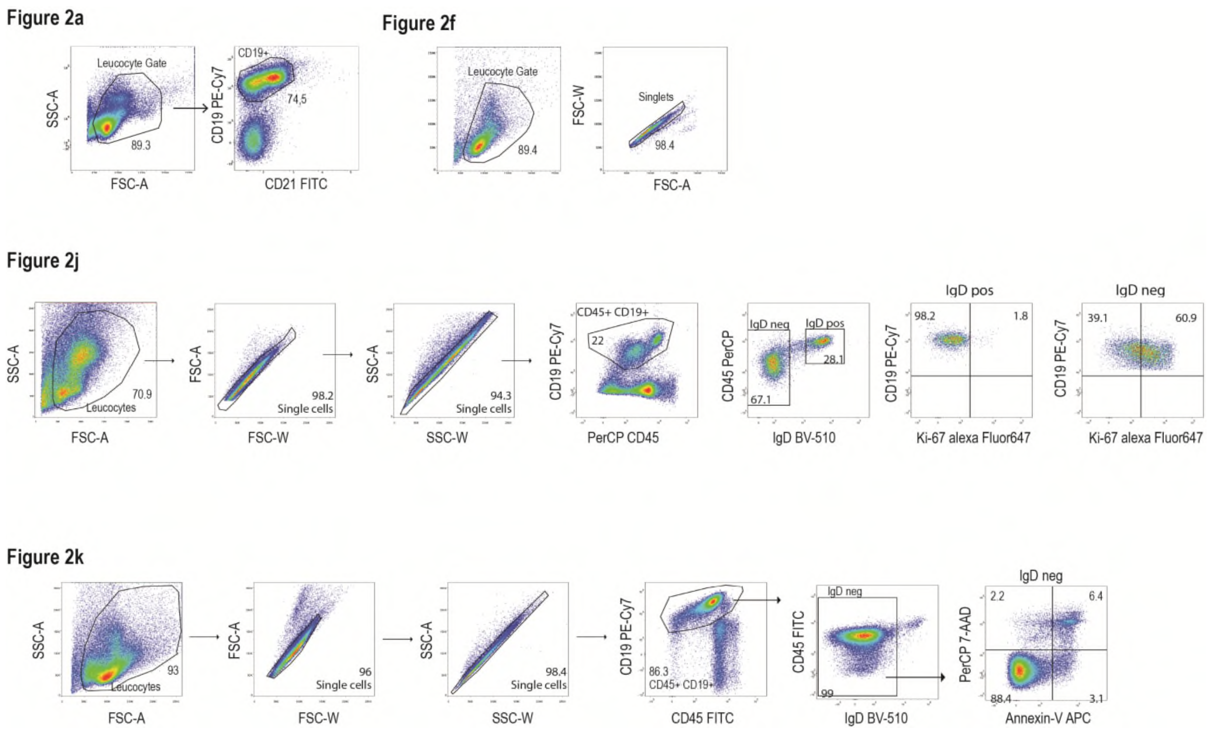

Supplementary Figure 27. Gating strategy for flow cytometry data in figure 2. Representative examples of the gating strategies used in the flow cytometry data presented in the indicated figure panels. Numbers in plots correspond to the frequency in the respective gate.

Figure 4h

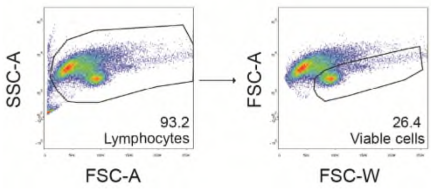

Supplementary Figure 28. Gating strategy for flow cytometry data in figure 4. Representative example of the gating strategy used in the flow cytometry analyses used to calculate the data presented in Fig. 4h. Numbers in plots correspond to the frequency in the respective gate.

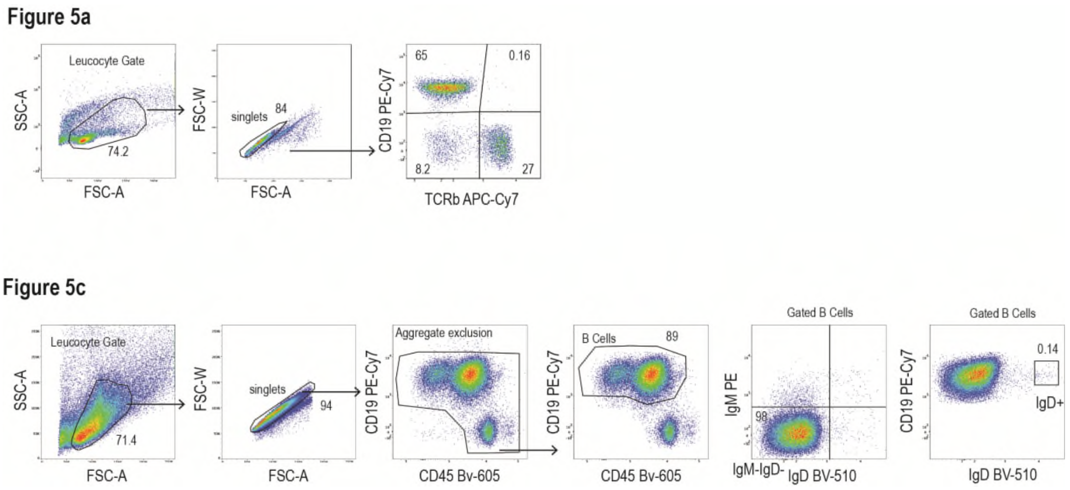

Supplementary Figure 29. Gating strategy for flow cytometry data in figure 5. Representative examples of the gating strategies used in the flow cytometry data presented in the indicated figure panels. Numbers in plots correspond to the frequency in the respective gate.

Figure 7b

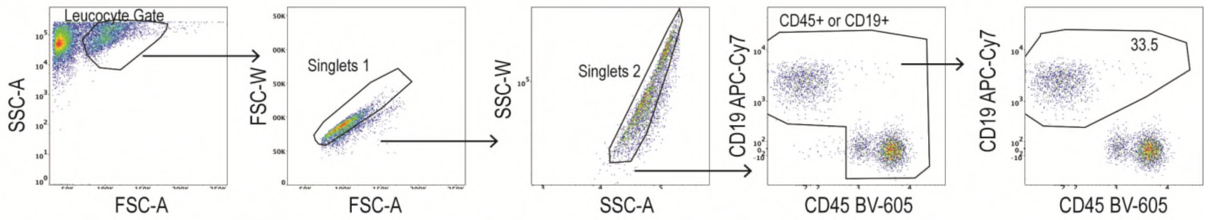

Figure 7g, h, i, S24 and S25

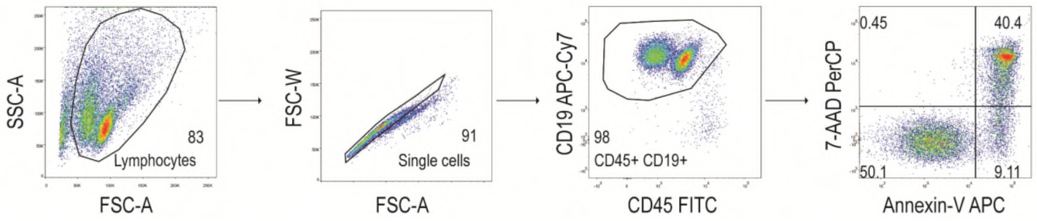

Supplementary Figure 30. Gating strategy for flow cytometry data in figure 7, supplementary figure 24 and supplementary figure 25. Representative examples of the gating strategies used in the flow cytometry data presented in the indicated figure panels. Numbers in plots correspond to the frequency in the respective gate.
