# Supplementary material for: Interleukin-7 receptor α mutational activation can initiate precursor B-cell acute lymphoblastic leukemia
Source: Nat Commun. 2021 Dec 14;12:7268. doi: 10.1038/s41467-021-27197-5 (PMC8671594; doi:10.1038/s41467-021-27197-5)
Supplement: Supplementary file 2 — Description of Additional Supplementary Files [file 41467_2021_27197_MOESM2_ESM.pdf]

## Description of Additional Supplementary Files

**Supplementary Data 1:** Immunophenotype and developmental stage of IL-7Rmut leukemias.

**Supplementary Data 2:** Productive and non-productive IG gene rearrangements based on WES data.

**Supplementary Data 3:** Ordered list of genes represented in figure 3a, showing differences between control and leukemia samples.

**Supplementary Data 4:** Complete list of RNAseq differentially expressed genes.

**Supplementary Data 5:** Ordered list of significantly differentially expressed proteins represented in figure 3b showing differences between control and leukemia samples.

**Supplementary Data 6:** Complete list of differentially expressed proteins from proteomics analysis.

**Supplementary Data 7:** PAM posterior probabilities for classification of mouse samples in B-ALL subtypes.

**Supplementary Data 8:** List of identified single nucleotide variants (SNVs) and indels in pre-leukemic, IL-7Rmut het leukemic and IL-7Rmut hom leukemic mouse samples.

**Supplementary Data 9:** List of identified copy number variants in pre-leukemic, IL-7Rmut het leukemic and IL-7Rmut hom leukemic mouse samples.

**Supplementary Data 10:** Profiler KEGG pathway functional enrichment analysis for the top 1000 genes contributing positively (related to subgroup Ph-like 2) and negatively (related to subgroup Ph-like 1) to the first principal component of the principal component analysis (PCA) presented in figure 6a.

**Supplementary Data 11:** PAM posterior probabilities for classification of human samples in Ph-like subgroups.

**Supplementary Data 12:** Absolute and relative frequencies of human B-ALL samples (from Gu et al, 2019), and B-ALL samples with disruptive exon 6 IL7R mutations within leukemia subtypes.

**Supplementary Data 13**: Z-score values of chemical screen library for kinase inhibitors tested in IL-7-dependent Ba/F3 cells stably transduced with mutant IL7R (Z-score cut-offs -17.59 and 7.79).

**Supplementary Data 14**: Antibodies used in flow cytometry analysis.

**Supplementary Data 15**: Primers used for Sanger sequencing.

**Supplementary Data 16**: Antibodies used in Immunoblotting.

**Supplementary Data 17:** Primer sequences.
