# Supplementary material for: Interleukin-7 receptor α mutational activation can initiate precursor B-cell acute lymphoblastic leukemia
Source: Nat Commun. 2021 Dec 14;12:7268. doi: 10.1038/s41467-021-27197-5 (PMC8671594; doi:10.1038/s41467-021-27197-5)
Supplement: Supplementary file 20 — Reporting summary [file 41467_2021_27197_MOESM20_ESM.pdf]

nature research

Corresponding author(s): J. Andres Yunes and João T. Barata

Last updated by author(s): 30/09/21

Reporting Summary

Nature Research wishes to improve the reproducibility of the work that we publish. This form provides structure for consistency and transparency in reporting. For further information on Nature Research policies, see Authors & Referees and the Editorial Policy Checklist .

Statistics

For all statistical analyses, confirm that the following items are present in the figure legend, table legend, main text, or Methods section.

n/a	Confirmed	
☐	☒	The exact sample size (*n*) for each experimental group/condition, given as a discrete number and unit of measurement
☐	☒	A statement on whether measurements were taken from distinct samples or whether the same sample was measured repeatedly
☐	☒	The statistical test(s) used AND whether they are one- or two-sided<br>*Only common tests should be described solely by name; describe more complex techniques in the Methods section.*
☒	☐	A description of all covariates tested
☐	☒	A description of any assumptions or corrections, such as tests of normality and adjustment for multiple comparisons
☐	☒	A full description of the statistical parameters including central tendency (e.g. means) or other basic estimates (e.g. regression coefficient) AND variation (e.g. standard deviation) or associated estimates of uncertainty (e.g. confidence intervals)
☐	☒	For null hypothesis testing, the test statistic (e.g. *F*, *t*, *r*) with confidence intervals, effect sizes, degrees of freedom and *P* value noted<br>*Give P values as exact values whenever suitable.*
☒	☐	For Bayesian analysis, information on the choice of priors and Markov chain Monte Carlo settings
☒	☐	For hierarchical and complex designs, identification of the appropriate level for tests and full reporting of outcomes
☒	☐	Estimates of effect sizes (e.g. Cohen's *d*, Pearson's *r*), indicating how they were calculated

Our web collection on statistics for biologists contains articles on many of the points above.

Software and code

Policy information about availability of computer code

Data collection	No software was used.
Data analysis	FlowJo v10, GraphPad Prism v6-8.05, RubioSeq v3.8a, Kallisto v0.44, FASTQC v0.11.7, MaxQuant version 1.6.0.1, samtools v 1.7, bedtools v2.27.1, GSEA v.4.0.3, R v3.4.4, R packages: edgeR v3.20.9, limma v3.34.9, CODEX v1.20.0, stats v3.4.4, preprocessCore v1.40.0, pamR v1.56.1, DEP v1.0.1., Modfit LT version 4

For manuscripts utilizing custom algorithms or software that are central to the research but not yet described in published literature, software must be made available to editors/reviewers. We strongly encourage code deposition in a community repository (e.g. GitHub). See the Nature Research guidelines for submitting code & software for further information.

Data

Policy information about availability of data

All manuscripts must include a data availability statement. This statement should provide the following information, where applicable:

- Accession codes, unique identifiers, or web links for publicly available datasets
- A list of figures that have associated raw data
- A description of any restrictions on data availability

The data supporting the findings from this study are available within the manuscript and the supplementary information. Source data are provided with this paper. In addition, WES and RNA-seq data are available from SRA under the accession number PRJNA718778 (https://www.ncbi.nlm.nih.gov/bioproject/PRJNA718778), and proteomics data are available on PRIDE under the accession number PXD017147 (http://proteomecentral.proteomexchange.org/cgi/GetDataset).

Field-specific reporting

Please select the one below that is the best fit for your research. If you are not sure, read the appropriate sections before making your selection.

☒ Life sciences ☐ Behavioural & social sciences ☐ Ecological, evolutionary & environmental sciences

For a reference copy of the document with all sections, see nature.com/documents/nr-reporting-summary-flat.pdf

Life sciences study design

All studies must disclose on these points even when the disclosure is negative.

Sample size	Animal experiments:<br>1-In order to evaluate the impact of aberrant IL7R (overexpression or mutant) expression in hematopoietic development, offspring littermates from different genotypes were analyzed at given ages in order to compare the relevant hematopoietic compartments (these are dependent on the exact Cre line). For these experiments, due to their fundamental nature and to the vast possibilities of relevant results, a power analysis is not always possible and thus we used the alternative "Resource equation" method, leading us to consider groups of 10 animals/ genotype/age in the calculations. Dividing query into specific questions (two-sided t-tests, assuming effect size 1.4-5, power 0,8 and 5% significance) calculations led to numbers/ group in the order of 9-10.<br>2- Transfer of leukemic cells into immune-deficient hosts to study leukemia expansion. In order to confirm leukemic nature of primary tumors. Leukemic cells recovered from disease carrying animals are recovered, sorted and transferred into secondary hosts, in order to confirm the capacity to transfer disease and phenotype stability and evaluate disease aggressiveness. For these experiments a minimal number of 3-4 animals was considered. Pilot experiments showed that all original leukemias (2x10e5 cells) were able to transfer disease into each recipient mice. Thus, we used the minimum number of animals that allowed to perform standard deviation analysis when required. Our subsequent experiments confirmed pilot data.<br>3- Transfer of leukemic cells into immune deficient hosts in order to study response to therapeutic agents. In these experiments, we try to test compounds in parallel whenever possible, in order to use same control groups and limit numbers of animals. We also performed power analysis for detecting effects, to detect a difference between means of 0.2 and, st deviation 0.15 and alpha 0.05 and power 0.8 we are calculating groups of 10 animals per condition. Our experience (Lonetti et al, Leukemia 2014) indicates we can achieve statistical significance with slightly lower numbers (>=6).<br>In order to answer questions related to leukemogenesis and associated molecular mechanisms, cohorts of offspring animals were followed in time, up to a limit of 104 weeks, evaluating penetrance and incidence in pediatric, adult and old age corresponding groups. Leukemias arising in these animals were a fundamental resource as source of leukemias for further dissection of molecular cooperating axis with the mutation/ aberration we introduced.<br><br>Experiments not involving animals: For other experiments, sample size was determined as according to our own experience on similar experimental designs (e.g. Zenatti et al, Nat Genet 2011; Silva et al, J Clin Inv 2008; Silva et al, Blood 2021) and/or available material (e.g. access to IL7R mutant primary ALL samples with sufficient biological material).
Data exclusions	In the leukemia incidence cohorts some animals become sick (mostly when >80 weeks of age) without evidence of leukemia in blood and were not considered in the analysis. In some of them, we could perform necropsy and confirm lack of signs for leukemia. Also, a very low number of animals was discarded from the analysis as result of handling accidents. Animals used in further breeding (to originate Homozygous mutant animals) were also not considered for the cohort. Results clearly state leukemia-free survival to account for spurious death not related to the experiment. In drug treatment, one animal died when administering anaesthesia, which was withdrawn from the data.
Replication	The experimental findings were reliably reproduced as validated by at least two independent experiments. In vitro experiments were performed in triplicate whenever possible.
Randomization	Randomization was performed in drug treatment experiments. At time of transfer, hosts were numbered, distributed into groups with web-based randomization tool (Graphpad Quickcalcs). In all other experiments, animals were compared with co-housed litter-mate controls.
Blinding	No blinding was performed, except for histological analysis that was performed blind by pathologists. Blinding was not required to group allocation during data collection and/or analysis since this is the standard approach for the experiments described in the manuscript, as there were no subjective measurements.

Reporting for specific materials, systems and methods

We require information from authors about some types of materials, experimental systems and methods used in many studies. Here, indicate whether each material, system or method listed is relevant to your study. If you are not sure if a list item applies to your research, read the appropriate section before selecting a response.

Materials & experimental systems

n/a	Involved in the study
	x Antibodies
	x Eukaryotic cell lines
x	Palaeontology
	x Animals and other organisms
	x Human research participants
x	Clinical data

Methods

n/a	Involved in the study
x	ChIP-seq
	x Flow cytometry
x	MRI-based neuroimaging

Antibodies

Antibodies used

Antibodies used are presented as supplementary data (supplemental table 14 and 16).

Validation

CD4 H129.19 FITC BioLegend 130308 1:100 https://www.biolegend.com/en-gb/products/fitc-anti-mouse-cd4-antibody-5487
IgM RMM-1 PE BioLegend 406508 1:100 https://www.biolegend.com/en-gb/products/pe-anti-mouse-igm-2332
IgM RMM-1 APC BioLegend 406509 1:50 https://www.biolegend.com/en-gb/products/apc-anti-mouse-igm-2335
IgM RMM-1 BV-605 BioLegend 406523 1:50 https://www.biolegend.com/en-gb/products/brilliant-violet-605-anti-mouse-igm-9616
Gr-1 RB6-8C5 PerCP BioLegend 108426 1:200 https://www.biolegend.com/en-gb/products/percp-anti-mouse-ly-6g-ly-6c-gr-1-antibody-4287
Gr-1 RB6-8C5 Biotin BioLegend 108404 1:200 https://www.biolegend.com/en-gb/products/biotin-anti-mouse-ly-6g-ly-6c-gr-1-antibody-457
CD19 6D5 PeCy7 BioLegend 115520 1:100 https://www.biolegend.com/en-gb/products/pe-cyanine7-anti-mouse-cd19-antibody-1907
CD19 6D5 APC BioLegend 115512 1:100 https://www.biolegend.com/en-gb/products/apc-anti-mouse-cd19-antibody-1526
CD19 6D5 APC-Cy7 BioLegend 115530 1:75 https://www.biolegend.com/en-gb/products/apc-cyanine7-anti-mouse-cd19-antibody-3903
CD19 6D5 BV-421 BioLegend 115538 1:100 https://www.biolegend.com/en-gb/products/brilliant-violet-421-anti-mouse-cd19-antibody-7160
CD19 6D5 Biotin BioLegend 115503 1:200 https://www.biolegend.com/en-gb/products/biotin-anti-mouse-cd19-antibody-1527
CD8 53-6.7 APC BioLegend 100712 1:100 https://www.biolegend.com/en-gb/products/apc-anti-mouse-cd8a-antibody-150
CD8 53-6.7 BV-605 BioLegend 100744 1:100 https://www.biolegend.com/en-gb/products/brilliant-violet-605-anti-mouse-cd8a-antibody-7636
TCRβ H57-597 APC-Cy7 BioLegend 109220 1:100 https://www.biolegend.com/en-gb/products/apc-cyanine7-anti-mouse-tcr-beta-chain-antibody-4137
IgD 11-26c.2a PerCP BioLegend 405736 1:100 https://www.biolegend.com/en-gb/products/percp-anti-mouse-igd-9574
IgD 11-26c.2a BV-510 BioLegend 405723 1:200 https://www.biolegend.com/en-gb/products/brilliant-violet-510-anti-mouse-igd-9032
CD45 30-F11 FITC BioLegend 103108 1:200 https://www.biolegend.com/en-gb/products/fitc-anti-mouse-cd45-antibody-99
CD45 30-F11 PerCP BioLegend 103130 1:100 https://www.biolegend.com/en-gb/products/percp-anti-mouse-cd45-antibody-4265
CD45 30-F11 BV-605 BioLegend 103140 1:200 https://www.biolegend.com/en-gb/products/brilliant-violet-605-anti-mouse-cd45-antibody-8721
CD11b M1/70 BV-711 BioLegend 101242 1:200 https://www.biolegend.com/en-gb/products/brilliant-violet-711-anti-mouse-human-cd11b-antibody-7927
CD11b M1/70 Biotin BioLegend 101203 1:200 https://www.biolegend.com/en-gb/products/biotin-anti-mouse-human-cd11b-antibody-346
BP-1 6C3 PeCy7 BioLegend 108314 1:100 https://www.biolegend.com/en-gb/products/pe-cyanine7-anti-mouse-ly-51-antibody-12284
CD127 A7R34 PE BioLegend 135010 1:100 https://www.biolegend.com/en-gb/products/pe-anti-mouse-cd127-il-7ralpha-antibody-6190
CD127 A7R34 BV-421 BioLegend 135024 1:100 https://www.biolegend.com/en-gb/products/brilliant-violet-421-anti-mouse-cd127-il-7ralpha-antibody-7193
CD25 PC61 PeCy7 BioLegend 102016 1:100 https://www.biolegend.com/en-gb/products/pe-cyanine7-anti-mouse-cd25-antibody-1929
CD25 PC61 BV-510 BioLegend 102042 1:100 https://www.biolegend.com/en-gb/products/brilliant-violet-510-anti-mouse-cd25-antibody-8663
CD24 M1/69 FITC BioLegend 101806 1:100 https://www.biolegend.com/en-gb/products/fitc-anti-mouse-cd24-antibody-341
CD93 AA4.1 APC BioLegend 136510 1:100 https://www.biolegend.com/en-gb/products/apc-anti-mouse-cd93-aa4-1-early-b-lineage-antibody-6621
B220 RA3-6B2 BV-711 BioLegend 103255 1:100 https://www.biolegend.com/en-gb/products/brilliant-violet-711-anti-mouse-human-cd45r-b220-antibody-9692
CD21 7 E 9 FITC BioLegend 123407 1:100 https://www.biolegend.com/en-gb/products/fitc-anti-mouse-cd21-cd35-cr2-cr1-antibody-4333
CD43 S7 PE BD Biosciences 561857 1:100 https://www.bdbiosciences.com/en-us/products/reagents/flow-cytometry-reagents/

research-reagents/single-color-antibodies-ruo/pe-rat-anti-mouse-cd43.561857
ckit 2B8 PeCy7 BioLegend 105814 1:100 https://www.biolegend.com/en-gb/products/pe-cyanine7-anti-mouse-cd117-c-kit-antibody-1900
CD5 53-7.3 APC-eFlour 780 eBioscience 47-0051-80 1:100 https://www.thermofisher.com/antibody/product/CD5-Antibody-clone-53-7-3-Monoclonal/47-0051-80
7AAD PerCP BD Biosciences 51-68981E/559925 1:50 https://www.bdbiosciences.com/en-pt/products/reagents/flow-cytometry-reagents/research-reagents/single-color-antibodies-ruo/7-aad.559925
Annexin V APC Invitrogen BMS306APC-100 1:100 https://www.thermofisher.com/antibody/product/Annexin-V-Recombinant-Protein/BMS306APC-100
KI67 16A8 Alexa Fluor 647 BioLegend 652408 1:400 https://www.biolegend.com/en-gb/products/alexa-fluor-647-anti-mouse-ki-67-antibody-8572
Isotype control RTK2758 Alexa Fluor 647 BioLegend 400526 1:400 https://www.biolegend.com/en-gb/products/alexa-fluor-647-rat-igg2a-kappa-isotype-ctrl-2683
FC Block 93 BioLegend 101302 1:50 https://www.biolegend.com/en-gb/products/purified-anti-mouse-cd16-32-antibody-190
CD44 IM7 PE BioLegend 103008 1:200 https://www.biolegend.com/en-gb/products/pe-anti-mouse-human-cd44-antibody-2206
CD44 IM7 PeCy7 BioLegend 103030 1:200 https://www.biolegend.com/en-gb/products/pe-cyanine7-anti-mouse-human-cd44-antibody-3932
CD3 17A2 APC BioLegend 100236 1:50 https://www.biolegend.com/en-gb/products/apc-anti-mouse-cd3-antibody-8055
CD3 17A2 BV-711 BioLegend 100241 1:50 https://www.biolegend.com/en-gb/products/brilliant-violet-711-anti-mouse-cd3-antibody-10022
NK1.1 PK136 APC eBioscience 17-5941-82 1:100 https://www.thermofisher.com/antibody/product/NK1-1-Antibody-clone-PK136-Monoclonal/17-5941-82
NK1.1 PK136 Biotin BioLegend 108704 1:100 https://www.biolegend.com/en-gb/products/biotin-anti-mouse-nk-1-1-antibody-428
CD11c N418 Biotin BioLegend 117304 1:150 https://www.biolegend.com/en-gb/products/biotin-anti-mouse-cd11c-antibody-1814
TER-119/Erythroid Cells TER-119 Biotin BioLegend 116204 1:100 https://www.biolegend.com/en-gb/products/biotin-anti-mouse-ter-119-erythroid-cells-antibody-1864
TCRGammaDelta GL3 BV-421 BioLegend 109230 1:100 https://www.biolegend.com/en-gb/products/brilliant-violet-421-anti-mouse-tcr-beta-chain-antibody-7251
Streptovidin BV-711 BioLegend 405241 1:100 https://www.biolegend.com/en-gb/products/brilliant-violet-711-streptavidin-9665
p-STAT5 (Y694) Cell Signaling Technology 9359L 1:1000 https://www.cellsignal.com/products/primary-antibodies/phospho-stat5-tyr694-c11c5-rabbit-mab/9359?site-search-type=Products&N=4294956287&Ntt=+9359l+&fromPage=plp&_requestid=766977
p-S6(S235/236) Cell Signaling Technology 2211S 1:1000 https://www.cellsignal.com/products/primary-antibodies/phospho-s6-ribosomal-protein-ser235-236-antibody/2211?site-search-type=Products&N=4294956287&Ntt=2211s&fromPage=plp&_requestid=766860
p-Akt (S473) Cell Signaling Technology 4060L 1:1000 https://www.cellsignal.com/products/primary-antibodies/phospho-akt-ser473-d9e-xp-rabbit-mab/4060?site-search-type=Products&N=4294956287&Ntt=+4060l+&fromPage=plp&_requestid=766811
STAT5 Cell Signaling Technology 94205S 1:2000 https://www.cellsignal.com/products/primary-antibodies/stat5-d2o6y-rabbit-mab/94205?site-search-type=Products&N=4294956287&Ntt=+94205s+&fromPage=plp&_requestid=766899
S6 Cell Signaling Technology 2217S 1:2000 https://www.cellsignal.com/products/primary-antibodies/s6-ribosomal-protein-5g10-rabbit-mab/2217?site-search-type=Products&N=4294956287&Ntt=2217s&fromPage=plp&_requestid=766761
Akt Cell Signaling Technology 9272S 1:2000 https://www.cellsignal.com/products/primary-antibodies/akt-antibody/9272?site-search-type=Products&N=4294956287&Ntt=9272s&fromPage=plp&_requestid=766734
β-actin Santa Cruz Biotechnologies SC-1616 1:10000 https://www.scbt.com/p/actin-antibody-i-19?requestFrom=search
Anti-Rabbit IgG HRP Conjugate Promega W4011 1:5000 https://worldwide.promega.com/products/protein-detection/primary-and-secondary-antibodies/anti-rabbit-igg-h-and-l-hrp-conjugate/?catNum=W4011
Anti-mouse IgG HRP conjugated Promega W4021 1:5000 https://worldwide.promega.com/products/protein-detection/primary-and-secondary-antibodies/anti_mouse-igg-h-and-l-hrp-conjugate/?catNum=W4021

Eukaryotic cell lines

Policy information about cell lines

Cell line source(s)	Parental Ba/F3 cells were obtained from Prof. Paul J. Coffer's lab, Utrecht University Medical Center, The Netherlands. HEK-293T cells were obtained from DSMZ.
Authentication	Parental Ba/F3 cells were confirmed for IL-3 dependence. Expression of mutant and wild type IL-7R was confirmed in transduced cells by flow cytometry and sequencing. Wild type IL7R-expressing cells were confirmed for IL-7 dependence. Mutant IL7R-expressing cells were confirmed for growth factor independence. HEK-293T cells were from commercial sources and thus not subsequently validated.
Mycoplasma contamination	Cell lines were routinely tested for Mycoplasma contamination, all cells used in the manuscript were tested negative.
Commonly misidentified lines (See ICLAC register)	None

Animals and other organisms

Policy information about studies involving animals; ARRIVE guidelines recommended for reporting animal research

Laboratory animals	Ill7rflCPT conditional knock-in animals in C57Bl/6 background were generated by Cyagen Biosciences (Santa Clara, CA). hCD2-iCre, B6 Rag-/- γc -/- and NSG animals were bred and kept at the IMM-JLA SPF animal facility. Male and female animals ranging in age from 4 to 104 weeks were used. For transfer experiments, 8-20 week old B6 Rag-/- γc -/- and NSG animals were used as hosts.
Wild animals	No wild animals were used.
Field-collected samples	No field collected samples in this article.
Ethics oversight	We identify the institutional and national organizations and commitees approving the study in the methods sections. IMM institutional and National (DGAV).

Note that full information on the approval of the study protocol must also be provided in the manuscript.

Human research participants

Policy information about studies involving human research participants

Population characteristics	We used 2 diagnostic samples from adult patients with precursor B-cell acute lymphoblastic leukemia and IL7R type 1a mutations, previously collected at Saint-Louis Hospital. Bone marrow mononuclear cells were isolated by Ficoll and cryopreserved in liquid nitrogen.
Recruitment	We used all the cryopreserved samples from patients that displayed IL7R type 1a mutations (as described in Barata et al, Nat Immunol 2019) that were available at Saint-Louis Hospital, Paris (n=2).
Ethics oversight	Patients were enrolled in GRAALL-2014 clinical trial (NCT02617004), approved by the French Comité de protection des personnes (CPP) and the Agence nationale de sécurité du médicament et des produits de santé (ANSM), and gave their informed consent for the use of their samples for research purposes.

Note that full information on the approval of the study protocol must also be provided in the manuscript.

Flow Cytometry

Plots

Confirm that:

- [x] The axis labels state the marker and fluorochrome used (e.g. CD4-FITC).
- [x] The axis scales are clearly visible. Include numbers along axes only for bottom left plot of group (a 'group' is an analysis of identical markers).
- [x] All plots are contour plots with outliers or pseudocolor plots.
- [x] A numerical value for number of cells or percentage (with statistics) is provided.

Methodology

Sample preparation	Blood was collected into tubes with heparin and red blood cells (RBCs) were lysed with lysis solution (Becton Dickinson San Jose, CA, USA), prior to staining with standard procedures. Splenic and thymic single cell suspensions were prepared by mashing over a filter and and BM single cell suspension were obtained by flushing and then immunophenotyped using standard methodology. Briefly, 10e6 cells were stained for 20 minutes at 4ºC in PBS with 2%FBS with specific antibodies. When lineage-positive cells were excluded, biotin coupled anti- Gr-1, CD11b, CD19, Ter119, NK1.1 and CD11c were used and subsequently stained with BV711 streptavidin. Proliferation was analyzed by intracellular staining of Ki67 (APC-conjugated, Biolegend), using the Foxp3 staining kit from eBioscience and following the manufacturer's instructions. Cell viability was determined using an annexin V-based apoptosis detection kit and following the manufacturer's instructions (eBioscience).
Instrument	LSR Fortessa II and FACSARia (Becton Dickinson San Jose, CA, USA).
Software	Results were analyzed with FlowJo (Tree StarInc., Ashland, OR, USA) software.
Cell population abundance	Sorted samples were >95% pure.
Gating strategy	Cell populations were gated on live cells, doublets were excluded and populations were defined as described in figure legends and in supplementary figures provided. Most relevant populations: B cells were defined as CD19+; T cells were defined as CD45 +CD3+ or TCR+ cells.

- [x] Tick this box to confirm that a figure exemplifying the gating strategy is provided in the Supplementary Information.
